# Characterization of Tau95 led to the identification of a four-subunit TFIIIC complex in trypanosomatid parasites

**DOI:** 10.1007/s00253-023-12903-8

**Published:** 2024-01-10

**Authors:** Fabiola Mondragón-Rosas, Luis E. Florencio-Martínez, Gino S. Villa-Delavequia, Rebeca G. Manning-Cela, Julio C. Carrero, Tomás Nepomuceno-Mejía, Santiago Martínez-Calvillo

**Affiliations:** 1 Facultad de Estudios Superiores Iztacala, Unidad de Biomedicina, Universidad Nacional Autónoma de México, Av. de los Barrios 1, Col. Los Reyes Iztacala, Tlalnepantla, Edo. de México CP 54090 México; 2 Departamento de Biomedicina Molecular, Centro de Investigación y de Estudios Avanzados del IPN, Av. IPN 2508, Ciudad de Mexico, CP 07360 México; 3 Departamento de Inmunología, Instituto de Investigaciones Biomédicas, Universidad Nacional Autónoma de México, Ciudad de Mexico, 04510 México

**Keywords:** *Leishmania major*, *Trypanosoma brucei*, RNAP III transcription, TFIIIC, Tau95, tRNA, 5S rRNA

## Abstract

**Abstract:**

RNA polymerase III (RNAP III) synthetizes small essential non-coding RNA molecules such as tRNAs and 5S rRNA. In yeast and vertebrates, RNAP III needs general transcription factors TFIIIA, TFIIIB, and TFIIIC to initiate transcription. TFIIIC, composed of six subunits, binds to internal promoter elements in RNAP III-dependent genes. Limited information is available about RNAP III transcription in the trypanosomatid protozoa *Trypanosoma brucei* and *Leishmania major*, which diverged early from the eukaryotic lineage. Analyses of the first published draft of the trypanosomatid genome sequences failed to recognize orthologs of any of the TFIIIC subunits, suggesting that this transcription factor is absent in these parasites. However, a putative TFIIIC subunit was recently annotated in the databases. Here we characterize this subunit in *T. brucei* and *L. major* and demonstrate that it corresponds to Tau95. In silico analyses showed that both proteins possess the typical Tau95 sequences: the DNA binding region and the dimerization domain. As anticipated for a transcription factor, Tau95 localized to the nucleus in insect forms of both parasites. Chromatin immunoprecipitation (ChIP) assays demonstrated that Tau95 binds to tRNA and U2 snRNA genes in *T. brucei*. Remarkably, by performing tandem affinity purifications we identified orthologs of TFIIIC subunits Tau55, Tau131, and Tau138 in *T. brucei* and *L. major*. Thus, contrary to what was assumed, trypanosomatid parasites do possess a TFIIIC complex. Other putative interacting partners of Tau95 were identified in *T. brucei* and *L. major*.

**Key points:**

• *A four-subunit TFIIIC complex is present in T. brucei and L. major*

• *TbTau95 associates with tRNA and U2 snRNA genes*

• *Putative interacting partners of Tau95 might include some RNAP II regulators*

**Supplementary Information:**

The online version contains supplementary material available at 10.1007/s00253-023-12903-8.

## Introduction

*Trypanosoma brucei* and *Leishmania major* are protozoan parasites that belong to the Trypanosomatid family. *T. brucei* is the causative agent of human African Trypanosomiasis, also known as sleeping sickness, in Sub-Saharan Africa. The parasite is transmitted to humans through the bite of the tsetse fly (*Glossina* spp.). *T. brucei* also causes the nagana disease in cattle, producing significant economic loss in the region (Pays et al. 2023). *L. major* is the etiological agent of cutaneous leishmaniasis in Central Asia, the Middle East, and Northern and Western Africa. This microorganism is spread to humans by the bite of infected female sandflies of the genus *Phlebotomus*. Cutaneous leishmaniasis is the most prevalent clinical leishmanial manifestation, showing around 800,000 new cases annually worldwide (de Vries and Schallig 2022). In addition to their medical relevance, trypanosomatid parasites are important in the molecular biology field because they possess unique mechanisms for gene expression, including polycistronic transcription of protein-coding genes, and messenger RNA (mRNA) processing by trans-splicing (Martínez-Calvillo et al. 2010; Clayton 2013).

In eukaryotic cells, transcription of nuclear DNA is carried out by RNA polymerases (RNAP) I, II, and III (Roeder 2019). While RNAP I specializes in the synthesis of ribosomal RNAs (rRNAs) 18S, 5.8S, and 28S, RNAP II transcribes multiple types of RNA molecules, including mRNAs, most small nuclear RNAs (snRNAs), and small nucleolar RNAs (snoRNAs) (Grummt 2003; Liu et al. 2013). RNAP III produces small essential RNA molecules, such as transfer RNAs (tRNAs), 5S rRNA, U6 snRNA, and 7SL RNA, which play key roles in several cellular processes that include protein synthesis and mRNA processing. Most RNAP III promoter regions fall into three different categories (Dieci et al. 2007; Leśniewska and Boguta 2017). Type I promoters are present in 5S rRNA genes, and consist of three internal domains: box A, an intermediate element, and box C. Type II promoters are characteristic of tRNA genes, and are composed of two conserved internal elements: boxes A and B. Type III promoters, typical of U6 snRNA genes, consist of elements located upstream of the coding sequence: a TATA box, a proximal sequence element, and a distal sequence element (Dieci et al. 2013).

In yeast and vertebrates, RNAP III requires three general transcription factors to initiate RNA synthesis: TFIIIA, TFIIIB, and TFIIIC (Geiduschek and Kassavetise 2001). All three general transcription factors are needed for transcription of 5S rRNA genes, whereas tRNA synthesis only requires TFIIIB and TFIIIC. Transcription of U6 snRNA genes involves TFIIIB and an additional factor called SNAPc. During the formation of the preinitiation complex in 5S rRNA and tRNA genes, TFIIIC binding to the internal promoter region is followed by the association of TFIIIB to the region located upstream of the transcription start site and the recruitment of RNAP III (Graczyk et al. 2018). In yeast, TFIIIC is a large (500 kDa) six-subunit complex organized into two subcomplexes called τA and τB, which specifically bind boxes A and B, respectively, in tRNA genes. The τA subcomplex is formed by Tau131, Tau95, and Tau55, whereas τB is composed of Tau138, Tau91, and Tau60 (Acker et al. 2013; Male et al. 2015).

In the τA subcomplex, Tau95 (known as Sfc1 in *Schizosaccharomyces pombe* and TFIIIC63 in human) is the only subunit with DNA binding properties, interacting directly with box A (Taylor et al. 2013a). Accordingly, Tau95 is characterized by the presence of a DNA binding domain, as well as a dimerization motif to bind to Tau55. The DNA binding domain is a compact region in the C-terminus of the protein that is formed by two subdomains: a canonical winged-helix domain fold with a α1-β1-α2-α3-β2-β3 topology, with an additional β-strand; and a unique fold that consists of five α-helices and two β-strands, named winged-helix interacting region (Taylor et al. 2013a). The dimerization domain, located in the N-terminus of Tau95, interacts with Tau55 to form a module composed of three interwoven β barrels containing 15 β-strands and four α-helices. In addition to Tau55, Tau95 establishes interactions with Tau131, the third τA subunit, and with subunits Tau138 and Tau91 from τB. For that reason, Tau95 has been proposed to act as a molecular linker between the two subcomplexes, contributing to the stability of the entire TFIIIC complex. Also, Tau95 has an indirect effect on transcription start site selection, as it interacts with Tau131, the protein directly involved in TFIIIB recruitment, whose positioning helps to determine the transcription start site (Jourdain et al. 2003; Male et al. 2015).

In trypanosomatid parasites, little is known about the proteins that help to regulate RNAP III transcription initiation. As in other species, in trypanosomatids, TFIIIB is composed of three subunits: the TATA-binding protein (TBP), Brf1 and Bdp1. In *T. brucei*, TBP is essential for transcription of all three RNAP (Ruan et al. 2004), and it has been shown to interact with tRNA and snRNA genes in *Leishmania* (Thomas et al. 2006, 2009). In *T. brucei* and *L. major*, Brf1 is a nuclear protein that associates with RNAP III genes and is essential for cell viability (Vélez-Ramírez et al. 2015; Florencio-Martínez et al. 2021). Similarly, Bdp1 is an essential protein needed for RNAP III transcription of tRNAs, snRNAs, and 5S rRNA in *L. major* (Román-Carraro et al. 2019). Other proteins that participate in the regulation of RNAP III transcription in trypanosomatids are Maf1 (Romero-Meza et al. 2017) and the SNAP50 subunit of SNAPc (Thomas et al. 2009).

Unlike TFIIIB, neither TFIIIA nor any TFIIIC subunit were initially identified in the Trypanosomatid genome databases, strongly suggesting that these transcription factors were absent in this early branched group of eukaryotic microorganisms (El-Sayed et al. 2005). However, a putative ortholog of subunit Tau131 was recently co-purified with the Brf1 subunit of TFIIIB by tandem affinity purification experiments in *L. major* (Florencio-Martínez et al. 2021). Moreover, similar experiments performed with the C82 subunit of RNAP III allowed the identification of a putative ortholog of subunit Tau95 in *T. brucei* (Martínez-Calvillo et al. manuscript in preparation), which was recently annotated as a putative TFIIIC subunit in the trypanosomatid databases. In this work we characterized Tau95 in *T. brucei* (TbTau95) and *L. major* (LmTau95). Bioinformatic analyses showed that both proteins contain moderately conserved DNA binding domain and dimerization domains. Chromatin immunoprecipitation (ChIP) assays demonstrated the association of TbTau95 with RNAP III–dependent genes. Tandem affinity purification experiments with transgenic *T. brucei* and *L. major* followed by mass spectrometry and bioinformatic analyses showed the association of Tau95 with Tau131. Remarkably, we identified two additional subunits of TFIIIC, Tau55, and Tau138, demonstrating that, contrary to what was thought, a TFIIIC complex is indeed present in *T. brucei* and *L. major* parasites.

## Materials and methods

### Bioinformatic analyses

Sequences were obtained from the NCBI database (http://www.ncbi.nlm.nih.gov) and the TriTrypDB database (release 62) (http://tritrypdb.org/tritrypdb/). Sequence alignments were made with the ClustalΩ program (http://www.ebi.ac.uk/Tools/msa/clustalo/) and shaded manually. Domain identification and secondary structure predictions were generated with the Phyre2 program (http://www.sbg.bio.ic.ac.uk/~phyre2). Homology modeling was made with Phyre2, I-TASSER (https://zhanglab.ccmb.med.umich.edu/I-TASSER/), and SWISS-MODEL (http://swissmodel.expasy.org/interactive). Models were visualized and edited with the PyMol V 2.1.1 program (https://pymol.org/2/). The tetra-trico peptide repeats (TPR) were identified with the TPRpred program (https://toolkit.tuebingen.mpg.de/tools/tprpred). Hypothetical proteins identified by mass spectrometry were analyzed with the HHpred server (https://toolkit.tuebingen.mpg.de/tools/hhpred).

### *T. brucei* and *L. major* cell culture and transfection

Procyclic forms of *T. brucei* strain 29–13 (Wirtz et al. 1999) were cultured at 28 °C in SDM-79 medium supplemented with 10% fetal bovine serum (Life Technologies Corporation, Grand Island, NY, USA), 50 μg/ml hygromycin B (Sigma-Aldrich, Darmstadt, Germany) and 15 μg/ml G418 (Sigma-Aldrich, Darmstadt, Germany). Transfection by electroporation was performed as previously described (Vélez-Ramírez et al. 2015). Selection of transfectants was carried out with phleomycin (2.5 μg/ml) for RNAi or with blasticidin (10 μg/ml) for PTP (Prot C-TEV-Prot A) tagging (Schimanski et al. 2005). Clones were obtained by serial dilution in 96-well plates. RNAi induction was performed by adding doxycycline (2 μg/ml) to the medium. In parallel, the same cell line was grown in the absence of doxycycline (non-induced control). For growth curves, parasites were counted daily and diluted to 2 × 10^6^ cells/ml; cumulative cell density was plotted. *L. major* promastigotes from strain MHOM/IL/81/Friedlin (LSB-132.1) were grown in BM medium with 10% fetal bovine serum (Life Technologies Corporation, Grand Island, NY, USA) at 28 °C (Florencio-Martínez et al. 2021). Transfection with the episomal LmTau95-PTP vector was performed by electroporation as previously described (Florencio-Martínez et al. 2021). Clones were obtained by spreading transfected cells on plates containing 0.7% Seaplaque GTG agarose (FMC Bioproducts, Philadelphia, PA, USA) in BM medium with 50 μg/ml G418.

### Plasmid constructs

To obtain plasmid pZTbTau95 for RNAi assays, a 383-bp fragment from the TbTau95 gene (Tb927.10.980) was amplified with primers TbTau95RNAi-F (5’-GGATCCAAGCTTAATAAAAAGGCTGTGGCGTGC) and TbTau95RNAi-R (5’-CTCGAGAAGGCGTCATCACTCACATCA) and cloned into the pZJM vector (Wang et al. 2000), using the *Xho*I and *Bam*HI restriction sites. Prior to transfection the vector was linearized with *Not*I. To generate the pC-TbTau95-PTP vector for PTP-tagging, a 563-bp fragment from the C-terminal region of the TbTau95 gene was amplified with primers TbTau95PTP-ApaI-F (5’-GGGCCCCGTGACATTAGTCGCGTTCCC) and TbTau95PTP-NotI-R (5’-GCGGCCGCGCTCCGTCATCTGGATGCT). This fragment was cloned into the genome-integration pC-PTP-BLA vector (Schimanski et al. 2005) using the *Apa*I and *Not*I restriction sites. Before transfection the plasmid was linearized with the *Bsi*WI enzyme. To obtain plasmid pCold-TbTau95, the entire TbTau95 gene was amplified with primers Tb-Tau95-BamHI (5′-GGATCCATGTGTATTCCCCTCTCC) and Tb-Tau95-XbaI-R2 (5′-TCTAGACCCGTCATCTGGATGCTG), cloned into pGEM-T Easy and then subcloned into the *Bam*HI and *Xba*I restriction sites of the pCold1 expression vector (Takara Bio Inc., San Jose, CA, USA). To produce the pLmTau95-PTP vector, the complete LmTau95 gene (LmjF.21.1100) (without the terminal codon) was amplified by PCR with oligonucleotides LmTau95-XmaI-For (5´-ACCCGGGCCATGACCGCTCCACACGGC-3´) and LmTau95-XbaI-Rev (5´-CGGCGGACGACGACGACGAGTCTAGAC-3´). This fragment was cloned into the episomal pB6-PTP plasmid (Moreno-Campos et al. 2016) digested with *Xma*I and *Xba*I. All vectors were verified by sequencing.

### Western blot analysis

Whole-cell extracts were obtained as previously described (Florencio-Martínez et al. 2021). For western blots, 50 μg of protein were fractionated by 10% SDS-PAGE and blotted onto PVDF (polyvinylidene difluoride) membranes (Bio-Rad, Hercules, CA, USA). The membranes were first incubated with rabbit primary monoclonal anti-Prot C antibodies (Delta Biolabs, Boise, ID, USA) with a 1:3000 dilution, or polyclonal β-tubulin antibody (Thermo Scientific, Waltham, MA, USA) with a 1:1500 dilution; and then with a horseradish peroxidase (HRP)-conjugated secondary antibody and developed with the Immobilon Western Chemiluminescent HRP substrate (Merck-Millipore, Darmstadt, Germany). For TbTau95 detection, we used a polyclonal anti-TbTau95 antiserum with a 1:5000 dilution.

### Indirect immunofluorescence

To determine the subcellular localization of the PTP-tagged proteins, we performed indirect immunofluorescence assays as previously described (Vélez-Ramírez et al. 2015; Nepomuceno-Mejía et al. 2018). *L. major* and *T. brucei* transgenic parasites were fixed with 4% paraformaldehyde. To detect LmTau95-PTP, *L. major* cells were then incubated with a rabbit anti-Prot C antibody (Delta Biolabs, Boise, ID, USA). We used a mouse anti-LmNop56 antibody as nucleolar marker. Next, parasites were treated with a mixture of secondary anti-rabbit antibody conjugated with Alexa-Fluor 488 (Life Technologies Corporation, Grand Island, NY, USA) and anti-mouse antibody conjugated with Alexa Fluor 568 (Life Technologies Corporation, Grand Island, NY, USA). To reveal TbTau95-PTP, *T. brucei* cells were incubated with rabbit anti-Prot C antibody followed by secondary anti-rabbit antibody conjugated with Alexa-Fluor 488. DNA was stained with DAPI (4′,6-diamidine-2′-phenylindole dihydrochloride). Images were obtained with a Zeiss AxioImager A2 microscope and analyzed with the ZEN 2012 software (Blue edition) (Zeiss, Oberkochen, Germany).

### Northern blot analysis

To verify mRNA depletion, northern blot experiments were performed. For this, total RNA was extracted with the TRI reagent (Sigma-Aldrich, Darmstadt, Germany), and 20 μg were run in an agarose-formaldehyde denaturing gel and transferred to Hybond-N nylon membrane (GE HealthCare, Chicago, IL, USA). As a probe, we employed the 383-bp TbTau95 fragment cloned into the RNAi vector pZTbTau95, which was labeled with [α-32P]-dCTP using the High Prime DNA Labelling Kit (Roche, Basel, Switzerland). Membranes were hybridized with a solution of formamide 50%, saline-sodium citrate (SSC) buffer 5 × , sodium dodecyl sulfate (SDS) 0.2%, Denhardts 4 × and salmon sperm DNA (100 μg/ml) at 42 °C, and then washed to a final stringency of 0.1 × SSC and 0.1% SDS at 65 °C.

### Tandem affinity purifications and mass spectrometry analysis

To determine the proteins that interact with TbTau95 and LmTau95, we performed tandem affinity purification assays, using mid-log phase parasites from both LmTau95-PTP and TbTau95-PTP cell lines (3 L at 3–4 × 10^7^ cells/ml) as previously described (Florencio-Martínez et al. 2021). The eluted proteins were concentrated with Amicon Ultracel 3 K columns (Merck-Millipore, Darmstadt, Germany) and by evaporation in a vacuum concentrator and analyzed by SDS–PAGE and SYPRO Ruby (Invitrogen, Carlsbad, CA, USA) staining. Individual lanes from the gels were sliced into two pieces and proteins subjected to in-gel tryptic digestion prior to liquid chromatography–mass spectrometry/mass spectrometry (LC–MS/MS) at the Core Facility for Proteomics and Mass Spectrometry from Upstate Medical University (Syracuse, NY, USA). The collision-induced dissociation spectra were compared with the *T. brucei* and *L. major* protein database from the TriTrypDB page (http://tritrypdb.org/tritrypdb/).

### Chromatin immunoprecipitation assays

ChIP experiments were performed at least three times as described previously (Romero-Meza et al. 2017). Briefly, 1.2 × 10^8^ cells were cross-linked with 1% formaldehyde for 5 min at 37* °C*, and lysed with a Vibra-Cell VCX130 ultrasonic processor (Sonics, Newtown, CT, USA) (15 s on/off, 40% amplitude, for 5 min). Nuclei were pelleted and resuspended in sonication buffer (1% SDS (sodium dodecyl sulfate), 10 mM EDTA (ethylenediaminetetraacetic acid), and 50 mM Tris-HCl, pH 8.0, with 1 × protease inhibitors). Chromatin was sonicated with a BioRuptor UCD-200 (Diagenode, Denville, NJ, USA) (30 s on/30 s off, high intensity) for 40 cycles, to an average DNA size of around 200 to 500 bp. The sonicated material was pre-cleared with protein A/G plus-agarose beads (Santa Cruz Biotechnology, Dallas, TX, USA) by mixing for 1 h at 4 °C. Chromatin samples were incubated overnight at 4 °C with rabbit anti-Prot A antibody (Sigma-Aldrich, Darmstadt, Germany) or nonspecific rabbit immune serum as negative control. The protein–DNA complexes were incubated for 2 h with protein A/G plus-agarose beads and 20 μg of sonicated salmon sperm DNA, and then washed as previously described (Vizuet-de-Rueda et al. 2016). To reverse the cross-links, the samples were incubated with 200 mM NaCl at 65 °C overnight and then treated with RNase A and proteinase K. DNA was precipitated with sodium acetate and ethanol and quantified.

### Quantitative real-time PCR assays

The regions of DNA to which TbTau95 binds were identified by performing quantitative real-time PCR (qPCR) with 2 ng of the immunoprecipitated DNA. The reactions were performed in duplicate with the Platinum SYBR Green qPCR SuperMix-UDG kit (Invitrogen, Carlsbad, CA, USA), using optimized primers and conditions. Results were analyzed with the 2^−ΔΔCq^ method, as reported before (Vélez-Ramírez et al. 2015; Vizuet-de-Rueda et al. 2016), and represented as the fold enrichment over negative control precipitations. The upstream control region (UCR) of the rRNA promoter was amplified with primers 18SUSE5 (5’-CACCCTCAAGACCGTAGCTC) and 18SUSE3 (5’-ACCCGTCCCTTATCAACACA); and domains I/II with primers 18SProm5 (5’-CTGTGGGGAACACACAAACA) and 18SProm3 (5’-CCCTGTAGAGGGAAACACCA). The 18S rRNA gene (Tb927.2.1452) was amplified with oligonucleotides Lm-rRNA18S5' (5’-CGGCCTCTAGGAATGAAGG) and Lm-rRNA18S3' (5’- CCCCTGAGACTGTAACCTC); and the α-tubulin gene (Tb927.1.2340) with TubqFw (5’-GGGCTTCCTCGTGTATCA) and TubqRv (5’-GCTTGGACTTCTTGCCATAG). The promoter of the *SL* gene was amplified with oligonucleotides SL-promoter-F (5’- CTACCGACACATTTCTGGC) and SL-promoter-R (5’-GCTGCTACTGGGAGCTTCTCATACC); and the SL RNA intergenic region with primers SL-inter-F (5’-ATGGCTTATACGTGCTCGTTTCTCC) and SL-inter-R (5’-AGCAGACTTTAAAGCGCCTATATGTG). The 5S rRNA gene (Tb927.8.1381) was amplified with primers rRNA5S-5 ´ (5’-GTCGAGTACGACCACACTTG) and 5SrRNA-R1 (5’-GAGTACGGCACTCAGGGTT). The tRNA-Ala gene (Tb927.7.6821) was amplified with primers AlaqFw (5’-GGGGATGTAGCTCAGATGG) and AlaqRv (5’-TGGAGAAGTTGGGTATCGATC); and the tRNA-Arg gene (Tb927.8.2859) with primers ArgqFw (5’-GGTCTCGTGGCGCAATG) and ArgqRv (5’-CGATCCCGGCAGGACTC). The intergenic region upstream of the tRNA-Ala gene was amplified with primers InterAla5’ (5’-CACTCTCCCGAGAATCGAAG) and InterAla3’ (5’-TGGGTGTGGAGTCGACTTTT); and the intergenic region upstream of the tRNA-Arg gene with primers InterArg5’ (5’-GGCTGAAAATAGCGGAAGTG) and InterArg3’ (5’-GCTAGCCCGTGTCGTTAGTC). The U2 snRNA gene (Tb927.2.5680) was amplified with primers U2qFW (5’-CTCGGCTATTTAGCTAAGATCAAGT) and U2qRV (5’-CGGGACAGCCAACAGTTT); and its promoter region with primers U2Prom5’ (5’-CACAACCTGTAGTGGCGGTA) and TbU2R (5’-GCATATCTTCTCGGCTATT).

### RT-qPCR

To analyze the abundance of different transcripts after TbTau95 RNAi, RT-qPCR assays were performed. Briefly, 1 μg of total RNA from the induced and non-induced cultures was used as template for the first strand cDNA synthesis using the SuperScript One-Step RT-PCR System with Platinum Taq DNA Polymerase (Invitrogen, Carlsbad, CA, USA) and 50 ng of random hexamers (Invitrogen, Carlsbad, CA, USA) or 50 ng of nested (dT) primer (5'-CCTCTGAAGGTTCACGGATCCACATCTAGATTTTTTTTTTTTTTTTTTVN). The cDNA was analyzed by quantitative real-time RT-PCR (RT-qPCR) assays using the Platinum SYBR Green qPCR SuperMix-UDG kit (Invitrogen, Carlsbad, CA, USA). TbTau95 was amplified with primers TbTau95-RNAi-F (5’-GGATCCAAGCTTAATAAAAAGGCTGTGGCGTGC) and Tb-Tau95-R2 (5’-CTCCTCTGAGCCTCGCTT). The procyclin gene (Tb927.6.510) was amplified with primers Procyclin-5 (5’-ATGGCACCTCGTTCCCTTTA) and ProcqRv (5’-CTTTGCCTCCCTTCACGATAAC); and the TFIIB gene (Tb927.9.5710) with primers Tf2bqFw (5′-GAACAGGGAACGCACATTAG) and Tf2bqRv (5′-TTGTTGACTTTGGTCACTTCC). The 5S rRNA gene (Tb927.8.1381) was amplified with primers rRNA5S-5 ´ (5’-GTCGAGTACGACCACACTTG) and 5S rRNA-3' (5’-TGAGCCTGTGAGTGCTTAACTT). The tRNA-Ala, tRNA-Arg, and U2 snRNA genes were amplified with the primers previously mentioned.

### Generation of TbTau95 polyclonal antibody

The pCold-TbTau95 vector was transformed into *Escherichia coli* BL21 (DE3) competent cells (Thermo Scientific, Waltham, MA, USA). Induction of the TbTau95 recombinant protein (TbTau95r) expression was achieved with 1 mM isopropyl β-d-1-thiogalactopyranoside (IPTG) at 37 °C for 18 h. The TbTau95r protein was purified by affinity chromatography with Ni-Sepharose 6 Fast Flow matrix (GE Healthcare, Chicago, IL, USA), according to the manufacturer’s instructions. Six-week-old male BALB/c mice were immunized intravenously with 100 μg of purified TbTau95r protein mixed with TiterMax Gold adjuvant (Sigma-Aldrich, Darmstadt, Germany) at a 1:1 (ratio). Pre-immune mouse serum was obtained before antigen inoculation. Blood samples were collected 6 weeks after antigen immunization, and anti-TbTau95 polyclonal serum was recovered by centrifugation. The specificity of the anti-TbTau95 polyclonal antibody was confirmed by the western blot analysis.

## Results

### The predicted structures of TbTau95 and LmTau95 are conserved

**Fig. 1:**
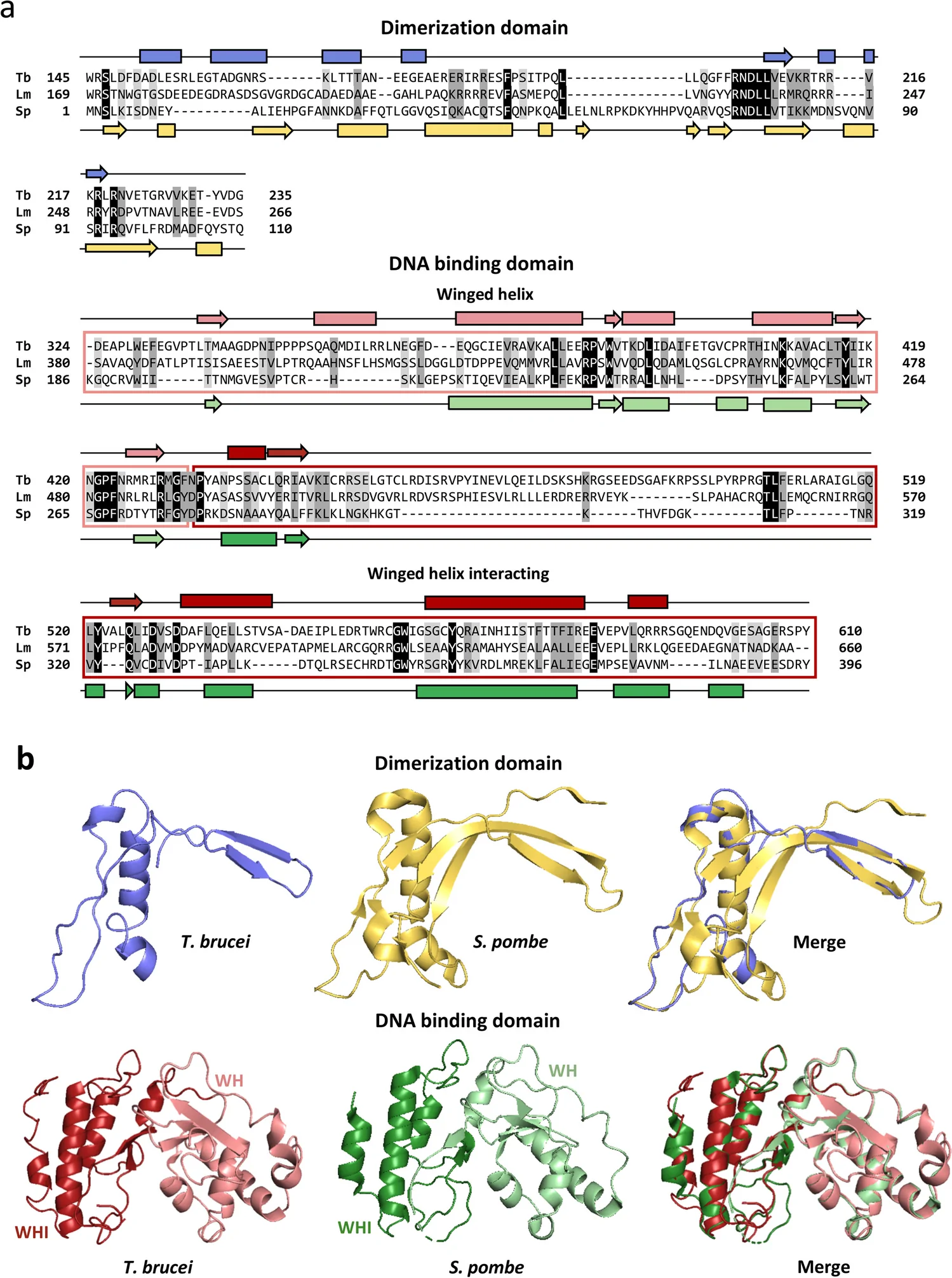
Sequence analysis and predicted three-dimensional structure of Tau95 in trypanosomatids. **a** Protein sequence alignment of the dimerization and DNA binding domains of Tau95 from *T. brucei* (Tb, Tb927.10.980), *L. major* (Lm, LmjF.21.1100), and *S. pombe* (Sp, NP_593297). Conserved residues are denoted by black shading, conserved substitutions are indicated by dark-gray shading, and semiconserved substitutions are denoted by light-gray shading, according to the Clustal Ω program. Predicted secondary structure elements are shown for *T. brucei* (above the sequence) and for *S. pombe* (below the sequence). The α-helices are indicated with rectangles and the β-strands with arrows. **b** Predicted three-dimensional structure of the dimerization and DNA binding domains of TbTau95 by homology modeling using the crystal structure of *S. pombe* Tau95 as a template. The structures are shown in the same colors presented in panel a. The quality of the models was estimated with Mod Eval server (https://modbase.compbio.ucsf.edu/evaluation/), showing a score of 0.70. The location of the winged helix (WH) and winged helix interacting (WHI) domains is indicated

*Tb927.10.980* and *LmjF.21.1100* were identified as the orthologs of *S. pombe* Tau95 (SpTau95) in *T. brucei* (TbTau95) (HHpred probability of 100%, E-value of 1.9 × 10^–40^) and *L. major* (LmTau95) (HHpred probability of 100%, E-value of 3.0 × 10^–40^), respectively (Martínez-Calvillo et al., manuscript in preparation). Amino acid sequence comparisons showed that TbTau95 and LmTau95 are 34.9% identical, and that they are 17.0 and 16.7% identical to SpTau95, respectively (Fig. 1a). These relatively low identities were somewhat expected, as multiple sequence alignments indicated that Tau95 is poorly conserved across evolution (Supplemental Fig. S1a). For instance, the identity between Tau95 from *Saccharomyces cerevisiae* and the orthologs in *S. pombe*, *Homo sapiens*, *Caenorhabditis elegans* and *Arabidopsis thaliana* are 24.0, 17.8, 19.3 and 21.0%, respectively (Supplemental Fig. S1a). Nevertheless, secondary and three-dimensional structure predictions, using the crystal structure of SpTau95 as a template, revealed that TbTau95 and LmTau95 contain the distinctive DNA binding and dimerization domains (Fig. 1), which are present in all Tau95 orthologs. Although some of the predicted secondary structures of the TbTau95 dimerization domain are not conserved (Fig. 1a), the overall three-dimensional structure is similar to that of SpTau95 (Fig. 1b). A higher conservation, both in secondary and three-dimensional structure, was observed in the winged-helix and winged-helix interacting subdomains of the DNA binding domain (Fig. 1). Thus, the in silico analyses show that the sequence and predicted models of Tau95 orthologs in trypanosomatids are relatively conserved. As anticipated, the sequence of Tau95 is highly conserved among *Leishmania* species (Supplemental Fig. S1b), with identities ranging from 80.1% (*Leishmania tarentolae* versus *Leishmania braziliensis*) to 99.5% (*Leishmania donovani* versus *Leishmania infantum*). The identity between Tau95 orthologs in *T. brucei* and *Trypanosoma cruzi* is 47.9% (Supplemental Fig. S1b).

### TbTau95 and LmTau95 are nuclear proteins

**Fig. 2:**
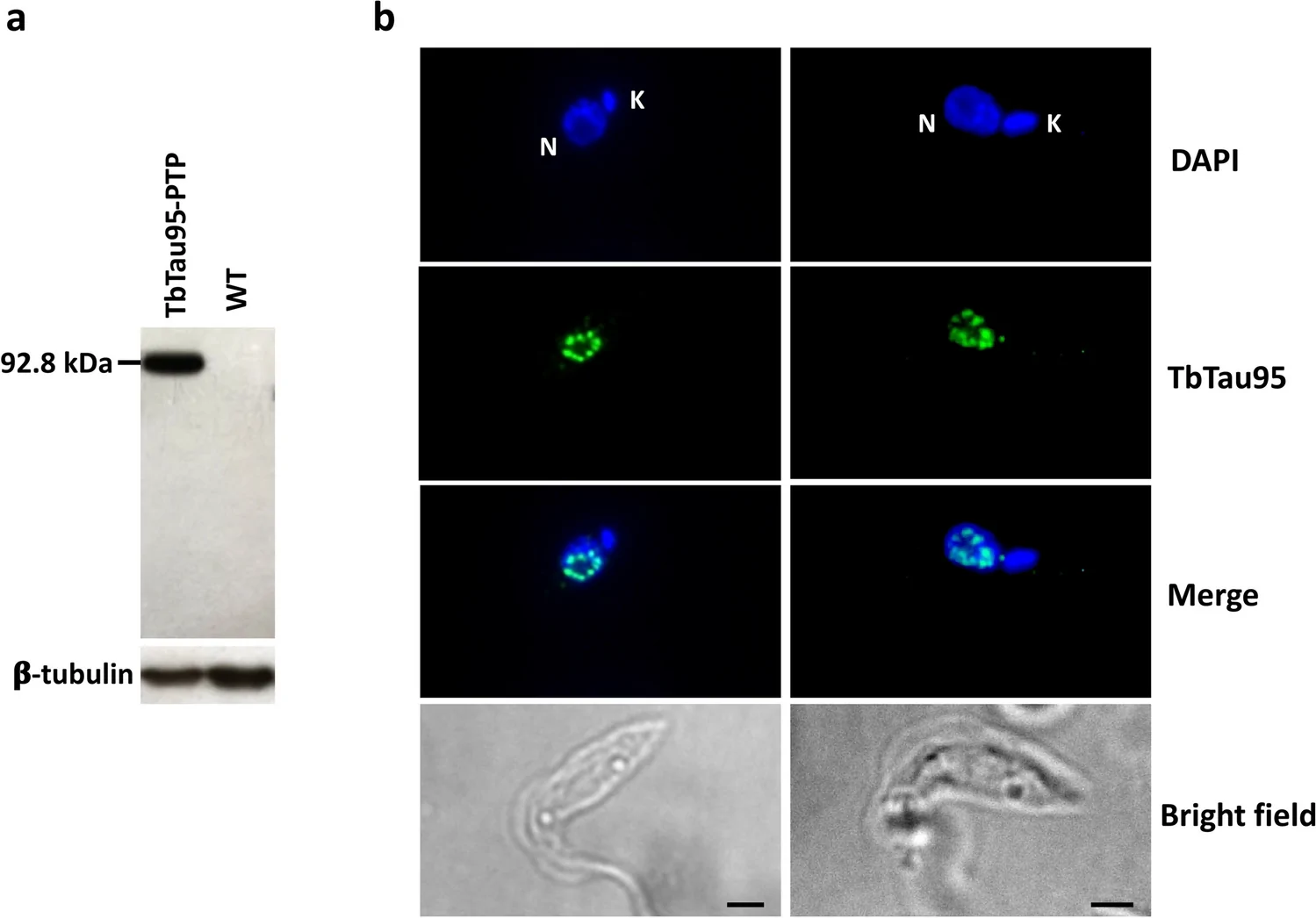
Nuclear localization of TbTau95. **a** Western blot analysis with total protein from wild-type (WT) parasites and transgenic cells that express the TbTau95-PTP protein using an anti-Prot C monoclonal antibody. β-tubulin was used as a loading control. **b** The location of TbTau95-PTP was determined by indirect immunofluorescence assays using anti-Prot C monoclonal antibody and an Alexa-Fluor 488 conjugated secondary antibody (Life Technologies Corporation, Grand Island, NY, USA). Nucleus (N) and kinetoplast (K) were stained with DAPI. Size bars represent 5 μm

To investigate the subcellular localization of TbTau95, we generated a cell line where the protein was tagged with a carboxy-terminal PTP tag to perform indirect immunofluorescence experiments. The PTP tag consists of Protein A (Prot A) and Protein C (Prot C) epitopes separated by a TEV protease cleavage site (Schimanski et al. 2005). To confirm the correct expression of the recombinant protein, western blot analysis was performed using a polyclonal antibody that recognizes the Prot C epitope, showing the expected protein of ~ 92.8 kDa (which resulted from the fusion of the 20 kDa PTP tag to the ~ 72.8 kDa TbTau95) (Fig. 2a). Indirect immunofluorescence assays carried out with the same antibody showed a dotted nucleoplasmic pattern that seems to surround the nucleolus (which is the nuclear region poorly stained with DAPI) of the transfected parasites (Fig. 2b).

**Fig. 3:**
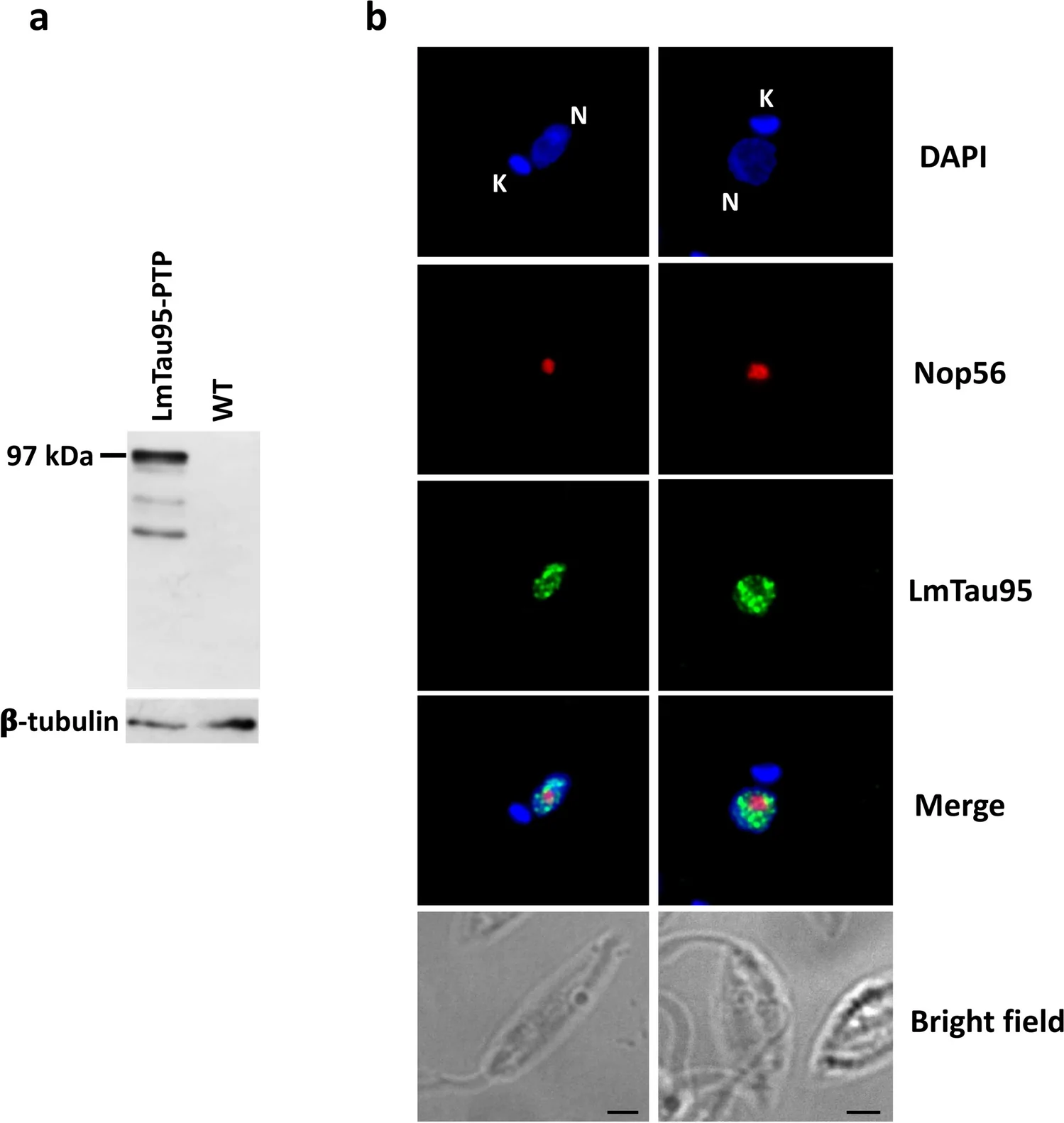
LmTau95 is a nuclear protein. **a** Western blot analysis with cells that express the recombinant protein LmTau95-PTP and wild-type (WT) cells. Membranes were incubated with an antibody against Prot C and an anti-β-tubulin antibody (loading control). **b** Indirect immunofluorescence experiments to determine the subcellular localization of LmTau95-PTP using an anti-Prot C antibody. As a nucleolar marker, an anti-LmNop56 antibody was used. Parasites were then treated with a mixture of secondary anti-rabbit antibody conjugated with Alexa-Fluor 488 and anti-mouse antibody conjugated with Alexa Fluor 568 (Life Technologies Corporation, Grand Island, NY, USA). Nucleus (N) and kinetoplast (K) were stained with DAPI. Size bars represent 5 μm

To determine the subcellular location of LmTau95, transgenic promastigotes that express this protein fused to a carboxy-terminal PTP epitope were produced. Expression of the LmTau95-PTP protein was confirmed by western blot with the anti-Prot C antibody, revealing the expected ~ 97 kDa band (the predicted mass of LmTau95 is ~ 76.5 kDa) (Fig. 3a). Indirect immunofluorescence experiments indicated that LmTau95-PTP localizes mainly to the nucleoplasm, with some weak signal observed in the nucleolus (Fig. 3b). Thus, our results showed that, as anticipated for a transcription factor, Tau95 is a nuclear protein in *T. brucei* and *L. major*.

### Attempts to knockdown TbTau95 were not successful

**Fig. 4:**
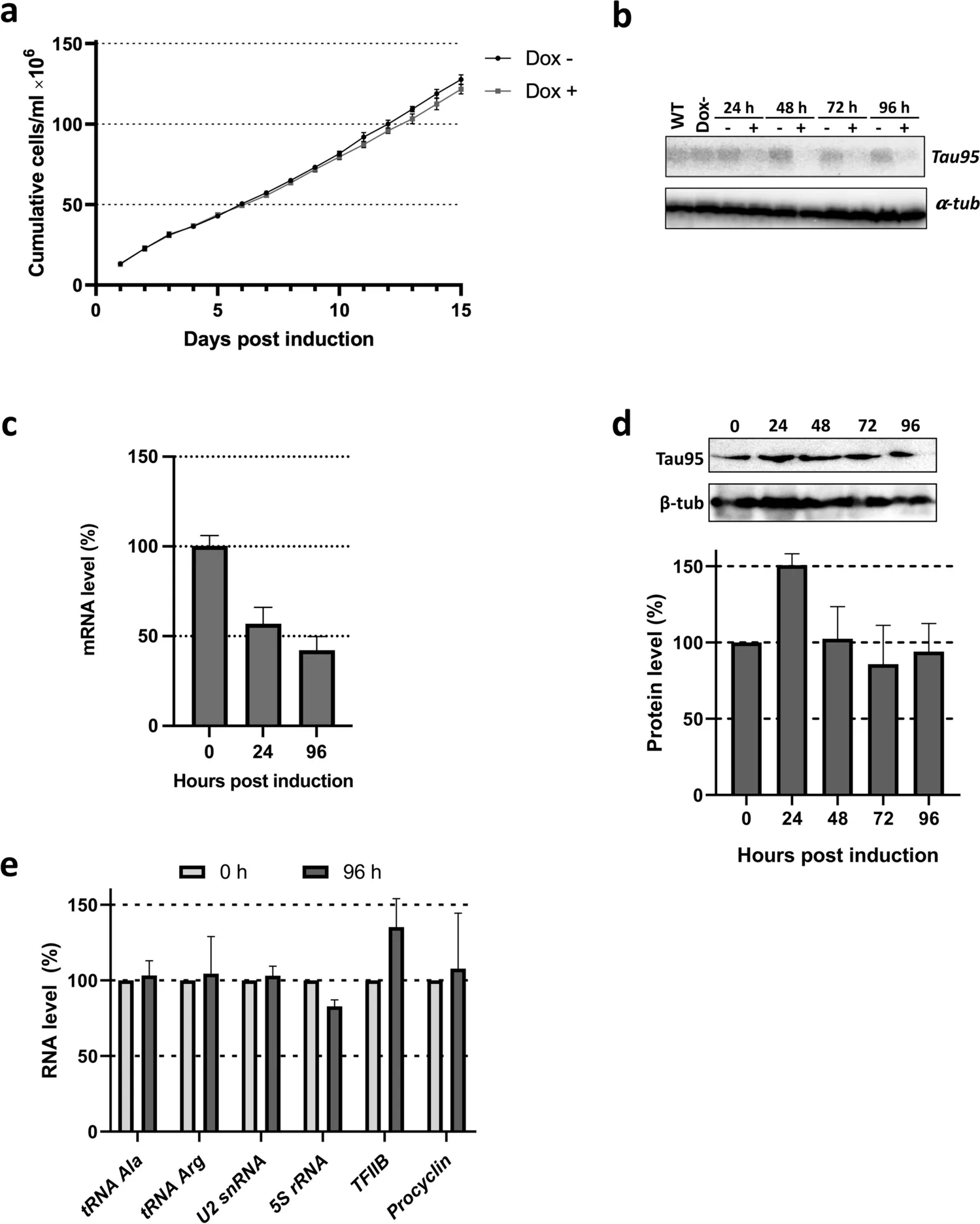
Attempt to knockdown TbTau95 by RNAi. **a** Growth curve of a clonal cell line obtained with the pZJM vector under non-induced (Dox−) and doxycycline-induced (Dox+) conditions. Cells were counted daily and diluted to a density of 2 × 10^6^ cells/ml. The values represent the cumulative cell density multiplied by the dilution factor. Data points reflect the means of triplicate experiments. Standard deviation bars are shown. **b** Northern blot analysis of TbTau95 mRNA in non-induced cells (Dox−), and cells induced for 24, 48, 72, and 96 h. RNA from wild-type (WT) cells was also analyzed. As a loading control, the filter was stripped and re-hybridized with an α-tubulin probe. **c** RT-qPCR experiments to determine the relative abundance of TbTau95 mRNA in non-induced cells (0 h) and cells induced for 24 and 96 h. **d** Western blot analysis of TbTau95 protein in non-induced cells (0 h), and cells induced for 24, 48, 72, and 96 h using a specific anti-TbTau95 polyclonal antibody at 1:5000 dilution. The bands shown here and from two independent experiments were quantified and plotted, considering as 100% the protein level obtained in the non-induced culture. Values represent means of the three experiments. Error bars indicate standard errors. TbTau95 protein levels were normalized to the level of the β-tubulin protein (loading control). **e** RT-qPCR assays to determine the relative abundance several transcripts in non-induced cells (0 h) and cells induced for 96 h. The transcripts analyzed were tRNA-Ala, tRNA-Arg, U2 snRNA, 5S rRNA, and the mRNAs from TFIIB and Procyclin. Error bars indicate standard errors

To assess whether TbTau95 is necessary for viability of procyclic forms of *T. brucei* in culture, we generated a cell line in which the knockdown of the protein by RNAi could be induced with doxycycline. To achieve this goal, a 383-bp fragment from the TbTau95 gene was cloned into the pZJM vector, which possesses two inducible T7 RNA polymerase promoters, in opposite direction, capable of generating double stranded RNA (dsRNA). This vector was transfected into a *T. brucei* cell line (29–13) that expresses the tetracycline repressor and T7 RNA polymerase (Wirtz et al. 1999). The resultant population was cloned by limiting dilution, and a clonal cell line was chosen for additional analysis. As shown in Fig. 4a, when comparing the growth of induced and non-induced cultures for 15 days, no significant differences were detected. Similar results were observed with a knockdown clone generated with the p2T7 RNAi vector (data not shown). To verify the depletion of TbTau95 mRNA after induction of RNAi with doxycycline, a northern blot experiment was carried out (Fig. 4b). Quantification of the signal showed that the Tau95 mRNA level was reduced by 91% after 48 h of induction. However, RT-qPCR experiments showed that the abundance of the Tau95 mRNA was only reduced by 58% after induction (Fig. 4c). In addition, western blot experiments with a TbTau95 polyclonal antiserum demonstrated that the amount of TbTau95 protein was not significantly reduced after doxycycline induction (Fig. 4d). Moreover, the abundance of several RNAP III-dependent transcripts was not affected in cultures induced for 4 days (Fig. 4e). Thus, these results indicate that the attempt to knockdown TbTau95 by RNAi was unsuccessful. It is worth noting that genome-wide RNAi knockdown screens in *T. brucei* indicate that TbTau95 is not an essential protein in procyclic and bloodstream forms of the parasite (Alsford et al. 2011).

### TbTau95 associates to several genomic regions

**Fig. 5:**
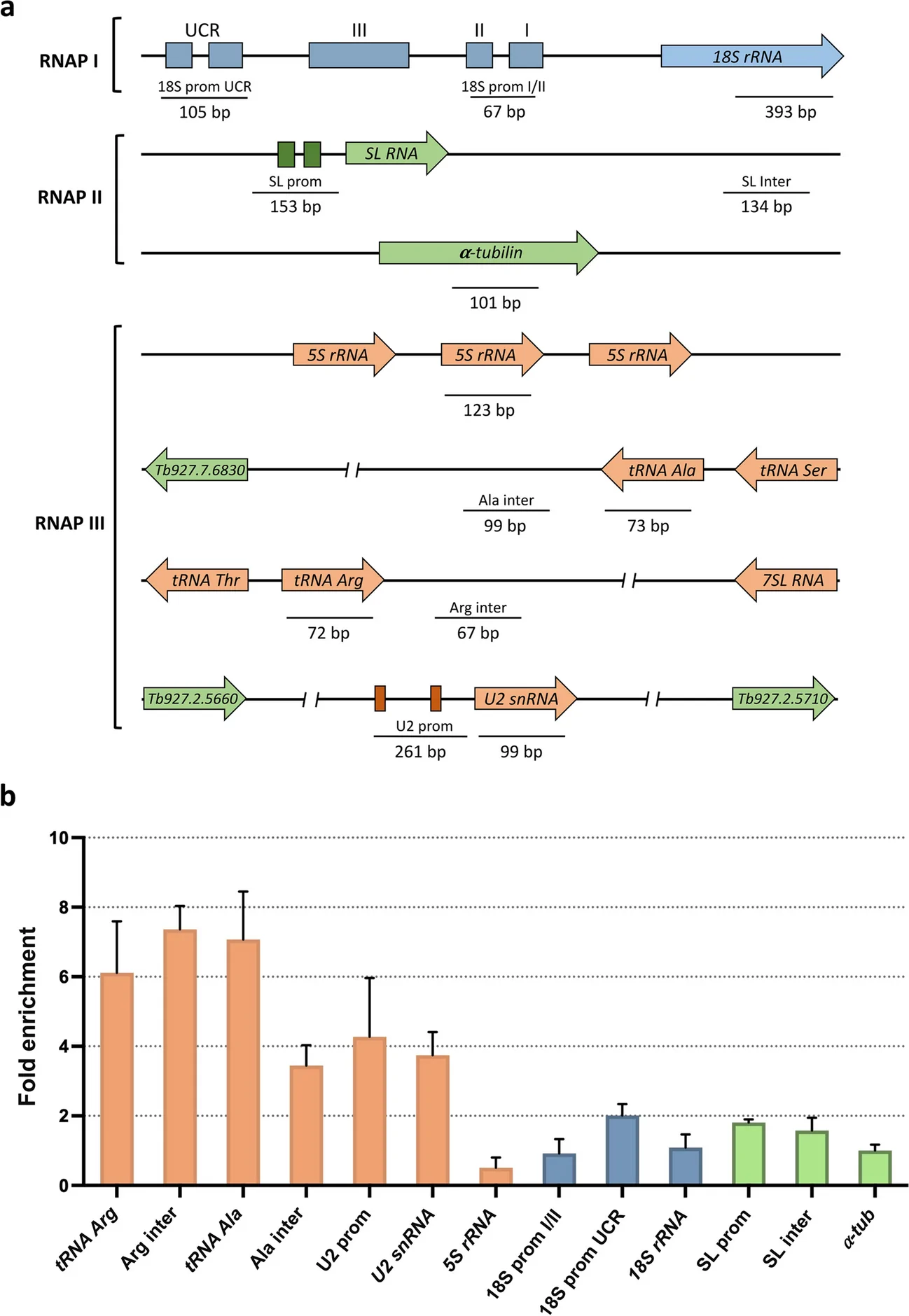
Chromatin immunoprecipitation analysis of TbTau95. **a** Schematic representation of the genes and promoter regions that were investigated. Genomic regions transcribed by RNAP III, RNAP II, and RNAP I are shown in orange, blue and green, respectively. **b** A ChIP grade anti-Prot A antibody was used to precipitate chromatin from the cell line that expresses the TbTau95-PTP protein. Precipitated DNA was examined by qPCR. The results from three independent ChIP experiments, each analyzed by two qPCR reactions, are shown. Error bars indicate standard deviations. Results are presented as fold enrichment over negative control precipitations

In order to explore whether TbTau95 binds to RNAP III-dependent genes and other genomic regions in vivo, ChIP assays were performed with the *T. brucei* transgenic line that expresses the recombinant TbTau95-PTP protein. These experiments were conducted in triplicate with a ChIP-grade anti-Prot A antibody that recognizes the two Prot A sequences located in the PTP tag. Chromatin was also precipitated with a nonspecific mouse immune serum, as a negative control. To evaluate the binding of TbTau95-PTP to the *T. brucei* genome, qPCR experiments were carried out with the purified DNA. The results showed high enrichment of TbTau95 in both tRNA genes analyzed and their flanking regions (Fig. 5). High association of TbTau95 was also observed in the U2 snRNA gene and its promoter region. Interestingly, no association of Tau95 to the 5S rRNA gene was found. Thus, except for the 5S rRNA gene, Tau95 associates strongly with RNAP III-dependent genes. Low occupancy of Tau95 was observed in the upstream control region of the rRNA promoter (transcribed by RNAP I) and the spliced-leader (SL) RNA gene promoter (transcribed by RNAP II) (Fig. 5).

### Trypanosomatids possess a TFIIIC complex

**Fig. 6:**
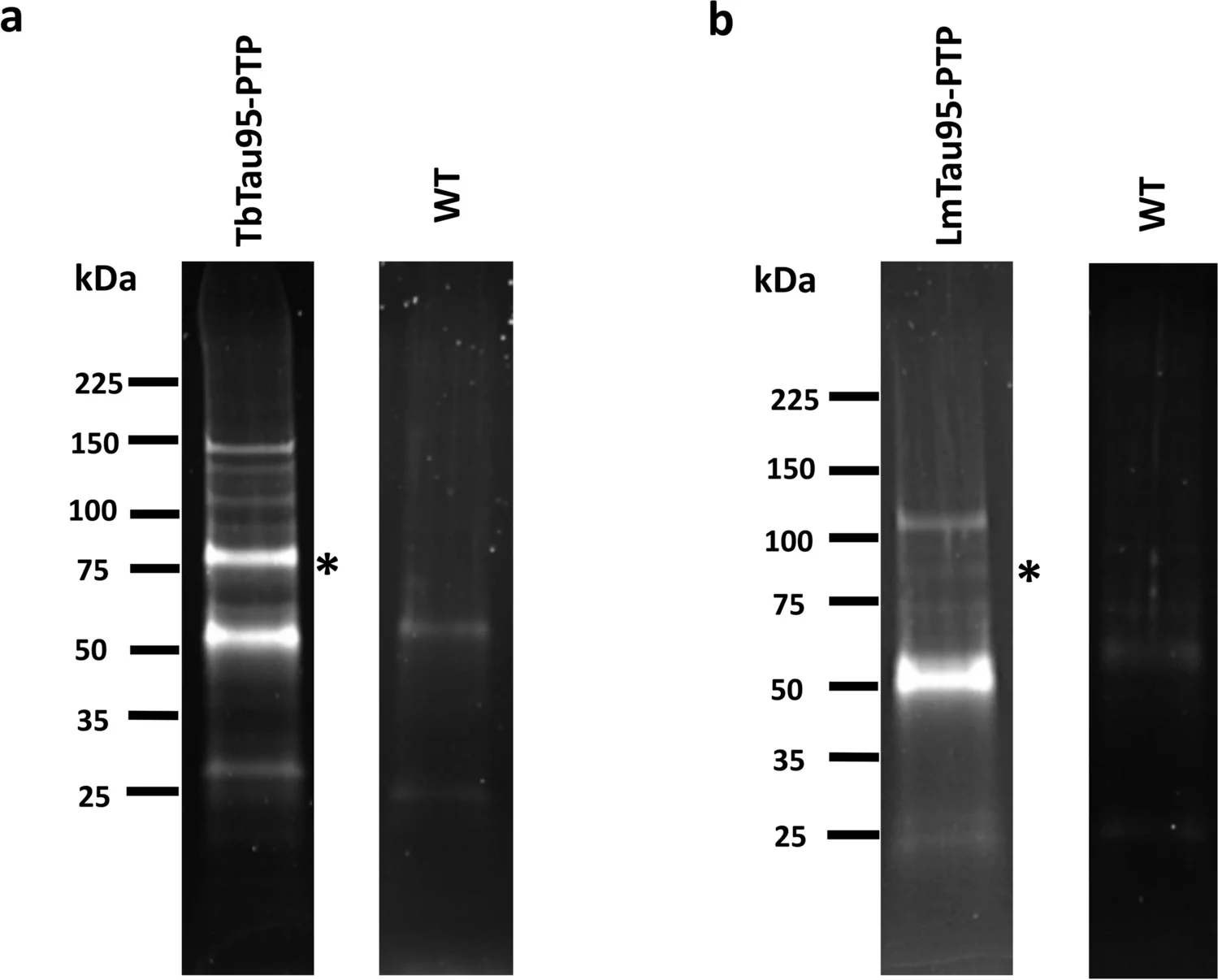
Tandem affinity purifications with parasites expressing Tau95-PTP recombinant proteins. SDS-PAGE of proteins copurified with TbTau95-PTP (**a**) and LmTau95-PTP (**b**). The asterisks indicate the recombinant proteins. Control experiments with wild-type (WT) *T. brucei* (**a**) and *L. major* (**b**) cells are also presented. Proteins were analyzed in 4–15% Mini- PROTEAN Precast Protein Gels (Bio-Rad, Hercules, CA, USA) stained with SYPRO Ruby (Invitrogen, Carlsbad, CA, USA)

To identify proteins that associate directly or indirectly with Tau95 in *T. brucei* and *L. major*, we performed tandem affinity purification experiments with the transgenic lines that express the recombinant proteins TbTau95-PTP and LmTau95-PTP, respectively. Interacting proteins were isolated by IgG affinity chromatography, TEV protease elution, and anti-Prot C affinity chromatography. SDS-PAGE analyses of the eluted proteins showed the presence of multiple bands, including two that could correspond to the PTP-tagged proteins after TEV digestion: a ~ 75-kDa band for TbTau95 (marked with an asterisk in Fig. 6a), and a ~ 77-kDa band for LmTau95 (denoted with an asterisk in Fig. 6b). As controls, mock purifications using wild-type *T. brucei* and *L. major* extracts were carried out. By performing mass spectrometry analyses with these controls, we identified proteins that are common contaminants in tandem affinity purifications (Mellacheruvu et al. 2013), such as bovine serum albumin, human keratins, and several trypanosomatid ribosomal proteins, heat-shock proteins, and other chaperons, translation elongation factors, mitochondrial proteins, α- and β-tubulins, and some other proteins (Supplemental Table S1).

Mass spectrometry and bioinformatic analyses of the LmTau95-PTP and TbTau95-PTP samples allowed the identification of multiple proteins (Supplemental Table S1). From the list, the proteins that were also identified in the wild-type controls were regarded as contaminants, together with other proteins that we have usually detected in unrelated purifications. Moreover, proteins that presented an average coverage of less than 20% in *T. brucei*, or less than 10% in *L. major*, were considered as possible contaminants (though we cannot exclude the possibility that some of them could represent bona fide interactors). A lower coverage threshold was set for *L. major* because in experimental replicate 2 the overall protein coverage was much lower than that found in replicate 1 (Table 2) and in both experiments with *T. brucei* (Table 1). Thus, after eliminating all these proteins, Tables 1 and 2 show the putative interacting partners of TbTau95 and LmTau95, which might associate directly or indirectly (via other proteins or RNA/DNA molecules) with the target proteins. Among the most abundant proteins that copurified with LmTau95-PTP, we found LmjF.12.0560 (Table 2), a tetratricopeptide repeat protein reported to interact with TFIIIB subunit Brf1, that was identified as the putative ortholog of yeast Tau131 (HHpred probability of 100%, *E* value of 1.1 × 10^−41^) (Florencio-Martínez et al. 2021). Notably, Tb927.1.3860, the *T. brucei* ortholog of LmjF.12.0560, copurified with TbTau95-PTP as well (Table 1). According to the HHpred server, the probability that Tb927.1.3860 corresponds to the yeast Tau131 ortholog is 100%, with an *E* value of 3.7 × 10^−56^. Thus, our results indicate that LmjF.12.0560 and Tb927.1.3860 are genuine orthologs of subunit Tau131 in *L. major* and *T. brucei*, respectively.

**Table 1:** Putative interacting partners of TbTau95

TriTrypDB name	Protein function (known or putative)	Predicted size (kDa)	Peptides^a^	Coverage (%)^b^
TFIIIC subunits				
Tb927.10.980	TFIIIC subunit Tau95	67.4	133, 111	72, 72
Tb927.11.4520	TFIIIC subunit Tau138	155.0	101, 116	58, 53
Tb927.1.3860	TFIIIC subunit Tau131	131.4	93, 58	53, 39
Tb927.11.1590	TFIIIC subunit Tau55	25.8	73, 63	84, 88
RNA polymerase subunits				
Tb927.2.2990	C82/RPC3 (RNAP III)	61.1	67, 51	76, 65
Tb927.10.15370	AC40/RPAC1 (RNAP I and III)	37.3	12, 7	36, 31
Regulators of transcription and/or chromatin remodelers				
Tb927.2.1080	Retrotransposon hot spot protein 5 (RHS5)	76.6	17, 13	27, 22
Tb927.4.2000	RuvB-like DNA helicase	52.6	12, 2	36, 5
Tb927.4.1270	RuvB-like DNA helicase	49.9	11, 7	35, 20
Tb927.2.4830	TFIIF-stimulated CTD phosphatase	38.8	10, 3	34, 9
DNA replication				
Tb927.11.5650	Replication factor C, subunit 1	65.0	13, 9	26, 21
Tb927.11.9550	Replication factor C, subunit 4	37.6	13, 1	56, 5
Tb927.6.3890	Replication factor C, subunit 2	38.8	11, 8	30, 28
Tb927.10.7990	Replication factor C subunit 3	38.7	9, 3	43, 14
RNA binding proteins				
Tb927.11.10550	Hypothetical protein (probable nucleic acid binding protein)	90.5	40, 25	45, 33
Tb927.6.4440	RNA-binding protein 42	37.7	5, 5	24, 17
Other functions				
Tb927.8.770	SUMO-interacting motif-containing protein, putative	70.7	57, 40	74, 67
Tb927.9.8880	Actin B	41.8	20, 6	65, 28
Tb11.v5.0718	Protein kinase	40.3	17, 3	56, 16
Tb927.6.4320	Protein of unknown function (DUF2817)	44.4	10, 5	35, 18

Proteins likely to be contaminants (including multiple ribosomal proteins, translation factors, tubulins, heat-shock proteins, mitochondrial proteins) were not included.

^a^Each digit indicates the number of peptides identified in two different tandem affinity purifications.

^b^Each number denotes the coverage found in two different experiments. Only proteins that show a coverage of at least 20%, in average, are shown

**Table 2:** Putative interacting partners of LmTau95

TriTrypDB name	Protein function (known or putative)	Predicted size (kDa)	Peptides^a^	Coverage (%)^b^
TFIIIC subunits				
LmjF.21.1100	TFIIIC subunit Tau95	76.5	183, 108	77, 63
LmjF.13.0270	TFIIIC subunit Tau138	207.3	49, 14	30, 10
LmjF.27.0990	TFIIIC subunit Tau55	29.9	37, 12	87, 29
LmjF.12.0560	TFIIIC subunit Tau131	149.0	39, 7	34, 7
RNA polymerase subunits				
LmjF.19.0660	AC40/RPAC1 (RNAP I and III)	47.4	9, 2	30, 9
LmjF.03.0790	C17/RPC9 (RNAP III)	45.4	3, 4	12, 16
Regulators of transcription and/or chromatin remodelers				
LmjF.29.1110	PAF1 complex novel subunit	68.5	10, 1	25, 3
LmjF.34.2610	RuvB-like DNA helicase	53.6	8, 2	21, 6
DNA replication				
LmjF.30.2630	Replication factor C, subunit 2	38.4	7, 1	34, 5
LmjF.24.0990	Replication factor C, subunit 1	71.7	7, 1	18, 2
Nucleolar proteins				
LmjF.05.0140	Nucleolar RNA helicase II	73.4	12, 1	18, 2
LmjF.10.0210	Nop56, rRNA processing	52.7	8, 2	25, 7
Other functions				
LmjF.34.2340	Asparaginyl-tRNA synthetase	99.4	11, 2	16, 4
LmjF.18.0700	HEAT repeats	77.3	10, 1	19, 3
LmjF.36.5880	Ras-like small GTPases	40.6	7, 1	27, 3
LmjF.31.2030	Ubiquitin-fusion protein	14.6	5, 2	37, 14
LmjF.04.1230	Actin	42.1	5, 1	25, 3

Proteins likely to be contaminants (including multiple ribosomal proteins, translation factors, tubulins, heat-shock proteins, and mitochondrial proteins) were not included

^a^Each digit indicates the number of peptides identified in two different tandem affinity purifications

^b^Each number denotes the coverage found in two different experiments. Only proteins that show a coverage of at least 10%, in average, are shown

Remarkably, putative orthologs of TFIIIC subunits Tau55 and Tau138 also copurified with LmTau95 and TbTau95. They are the most abundant proteins that copurified with both tagged proteins, together with Tau131 (Tables 1 and 2), and they all are annotated as hypothetical proteins in the TriTrypDB databases. LmjF.27.0990 (HHpred probability of 84.2%, *E* value of 1.5) and Tb927.11.1590 (probability of 82.9%, *E* value of 1.8) were identified as the feasible orthologs of *S. pombe* Tau55. Likewise, LmjF.13.0270 (probability of 30.2%, *E* value of 240) and Tb927.11.4520 (probability of 78.9%, *E* value of 13) are the putative orthologs of *S. cerevisiae* Tau138. Homologs of the missing TFIIIC subunits, Tau60 and Tau91, were not copurified with either LmTau95 or TbTau95. Thus, contrary to what was presumed, our results indicate that trypanosomatid parasites possess a TFIIIC complex, which seems to be composed of only four subunits.

### Proteins that participate in several functions copurified with TbTau95-PTP and LmTau95-PTP

In addition to TFIIIC subunits, several other proteins copurified with TbTau95-PTP and LmTau95-PTP, which were classified into the following categories: RNAP subunits, regulation of transcription and/or chromatin remodelers, DNA replication, RNA binding proteins, nucleolar proteins, and other functions (Tables 1 and 2). With both TbTau95 and LmTau95, we isolated subunit AC40, which is shared between RNAPs I and III. RNAP III specific subunits C82 and C17 copurified with TbTau95 and LmTau95, respectively. Among the putative regulator or transcription or chromatin remodelers we identified RuvB-like DNA helicases, the TFIIF-stimulated CTD phosphatase, and a PAF1 complex subunit. Several subunits of replication factor C were also identified (Tables 1 and 2).

## Discussion

Transcription factor TFIIIC has been extensively studied in organisms ranging from yeast to vertebrates (Dieci et al. 2013; Talyzina et al. 2023). Nevertheless, the knowledge about TFIIIC in early diverged eukaryotes is practically nonexistent. In this work, we characterize TFIIIC subunit Tau95 in the unicellular protozoan parasites *T. brucei* and *L. major*. Our results indicate that the amino acid sequence of Tau95 is weakly conserved across evolution (Supplemental Fig. S1a). However, these proteins contain a relatively conserved DNA binding domain, and a well conserved dimerization domain (Fig. 1). A stretch of acidic residues is conserved in the C-terminal region of Tau95 orthologs. In *S. pombe*, this acidic tail inhibits the DNA binding activity of Tau95 (Taylor et al. 2013a). Of note, both TbTau95 and LmTau95 possess the C-terminal acidic tail (Supplemental Fig. S2).

*T. brucei* and *L. major* cell lines that express the Tau95 protein fused to a C-terminal PTP tag were generated. Indirect immunofluorescence experiments with these cell lines showed that, as expected for a transcription factor, Tau95 is localized to the nucleus in both parasites. Interestingly, while a spotted ring-shaped pattern was observed in most *T. brucei* cells (Fig. 2b), Tau95 signal spreads throughout the nucleoplasm in *L. major* cells (Fig. 3b). A genome-wide study that mapped the subcellular localization of most *T. brucei* proteins also reported a nuclear localization for C-terminally GFP-tagged TbTau95, while an N-terminal tag promoted the relocalization of the protein to endosomes and cytoplasm (Billington et al. 2023) (data available in the TrypTag website, http://tryptag.org). Tagged Tau55 (Tb927.11.1590) and Tau138 (Tb927.11.4520) were found in the nucleus, although some signal was also observed in the cytoplasm. The study did not report the localization of Tau131 (Tb927.1.3860) (Billington et al. 2023).

Epitope tagging can potentially modify the function of the target protein. However, the following observations indicate that the general functions of the Tau95-PTP fusion proteins were not altered in the *T. brucei* and *L. major* transgenic cultures: (1) as anticipated for a transcription factor, immunofluorescence experiments show that the Tau95-PTP proteins localize to the nucleus; (2) ChIP assays demonstrate that TbTau95-PTP interacts with tRNA and snRNA genes, as expected for a TFIIIC subunit; and (3) tandem affinity purifications show that Tau95-PTP associates with other TFIIIC subunits and with RNAP III (see below). Nevertheless, the tag could have affected some specific functions of Tau95, such as its association with 5S rRNA genes (Fig. 5).

ChIP assays carried out with the TbTau95-PTP cell line showed high occupancy of TbTau95 in tRNA genes and their flanking regions (Fig. 5). Also, high enrichment of TbTau595 was found in the U2 snRNA gene and its upstream promoter (composed of A and B boxes located within a tRNA-like region). This is in agreement with the fact that, unlike other organisms, all snRNA genes are transcribed by RNAP III in trypanosomatids, and their expression is controlled by upstream tRNA (or tRNA-like) genes (Nakaar et al. 1994). Consequently, TFIIIC is expected to participate in the transcription of snRNA genes in these parasites. Interestingly, occupancy of TbTau95 was not observed in the 5S rRNA gene, as has been found in other organisms (Acker et al. 2013). Regarding RNAP I and II-dependent genes, low occupancy of TbTau95 was found in the rRNA promoter region and the SL RNA promoter (Fig. 5). It is worth noting that occupancy of subunit Brf1 of TFIIIB was observed in genes transcribed by RNAP I and II in *L. major* (Florencio-Martínez et al. 2021). Thus, it is possible that some RNAP III transcription factors participate in the regulation of global transcription in trypanosomatids. ChIP-Seq experiments would be needed to explore the potential association of TFIIIC and/or TFIIIB with RNAP I and II-dependent genes in trypanosomatids. Interestingly, TFIIIC was reported to associate to multiple RNAP II transcription start sites in neuroblastoma cell lines (Büchel et al. 2017).

Tandem affinity purification experiments with the TbTau95-PTP and LmTau95-PTP transgenic lines, followed by mass spectrometry and bioinformatic analyses, allowed us to identify orthologs of TFIIIC subunits Tau55, Tau131, and Tau138 in trypanosomatids (Tables 1 and 2). Tau55 exhibits only restricted sequence conservation across evolution, limited to a short region of 34 amino acids located within the dimerization domain with Tau95 (Dumay-Odelot et al. 2007). Our in silico analysis allowed the identification of this sequence in TbTau55 and LmTau55 (Supplemental Fig. S3). A histidine phosphatase domain, apparently not involved in RNAP III transcription, has been observed in the N-terminal region of Tau55 in *S. cerevisiae* and other hemiascomycetes, but not in other eukaryotes (Taylor et al. 2013b). Thus, as expected, the histidine phosphatase domain is not present in TbTau55 and LmTau55. Interestingly, the overall domain architecture of the Tau95-Tau55 heterodimer is very similar to that of subunits Rap30-Rap74 of the RNAP II general transcription factor TFIIF, as the dimerization module in both heterodimers consists of a triple β-barrel domain; and the C-terminal regions of Tau95 and Rap30 possess winged-helix domains as cryptic DNA binding domains (Taylor et al. 2013a).

Tau131 presents the highest structural conservation of the TFIIIC subunits, as it is mainly composed of tetra-trico peptide repeats (TPRs). The first 10 TPRs, in the N-terminal region, are clustered into two arrays: the left arm (TPRs 1–5) and the right arm (TPRs 6–10) (Male et al. 2015). The C-terminal region contains a helical domain and an extra TPR array with seven repeats (Vorlander et al. 2020). TbTau131 and LmTau131 are predicted to contain 10 and 12 TPR repeats in the N-terminal region, respectively, and six TPRs in the C-terminal region (Supplemental Fig. S3). Thus, the distinctive domain organization of Tau131 is conserved in trypanosomatids.

In the τB subcomplex, Tau138 tightly binds to the box B of tRNA genes. Despite this important role, Tau138 is the least well-characterized TFIIIC subunit (Male et al. 2015). Tau138 exhibits limited sequence conservation across eukaryotes, as the *S. cerevisiae* protein is related to the *S. pombe* ortholog, but not to the human one. While several winged-helix domains and a high mobility group motif are present in yeast Tau138 (Male et al. 2015), human Tau138 contains zinc finger and histone acetyltransferase domains (Vezzoli et al. 2023). TbTau138 and LmTau138 show similarity to a central extended winged-helix domain of *S. cerevisiae* Tau138 (Supplemental Fig. S3), which is the only region of the protein whose crystal structure has been determined. This domain is also moderately well-conserved between yeast and human, and is essential for yeast survival (Male et al. 2015).

We did not identify candidates for Tau60 and Tau91 orthologs in trypanosomatids. Both Tau60 and Tau91 are characterized by the presence of WD40 repeats (Mylona et al. 2006), which are commonly seven-bladed β-propeller domains with an overall doughnut shape that participate in protein-protein interactions (Schapira et al. 2017). It has been proposed that Tau60 and Tau91 form a platform for Tau138 interaction, thus regulating the binding of Tau138 to the box B (Mylona et al. 2006). Among the most abundant proteins that copurified with both TbTau95 and LmTau95 (that would represent the most obvious candidates for the missing subunits), we did not find proteins with homology to Tau60 and Tau91, and none of them are predicted to contain WD40 repeats. For instance, the hypothetical protein Tb927.11.10550, which was copurified with TbTau95 with a high number of peptides and coverage (Table 1), shows only weak homology to some nucleic acid binding proteins, but not to Tau60 or Tau91. A similar situation was observed with Tb927.8.770, annotated as putative SUMO-interacting motif-containing protein. Since these proteins do not show similarity to TFIIIC subunits or any other known transcription factor, they might represent trypanosomatid-specific regulators of transcription. Thus, our data strongly suggest that orthologs of Tau60 and Tau91 are absent in trypanosomatids. It is worth mentioning that an ortholog of TFIIIA, characterized by the presence of multiple zinc fingers, was not identified in our experiments either.

Several other proteins related to RNAP III transcription were identified by the mass spectrometry analyses with one or both parasites (Supplemental Table S1). These include RNAP III subunits, TFIIIB subunits Brf1 and TBP, and the transcriptional repressor Maf1 (although with low coverage, and therefore they are not shown in Tables 1 and 2). Interestingly, some proteins related to transcription by RNAP II were identified, representing potential interacting partners of TbTau95 and LmTau95 (Tables 1 and 2). Similarly, proteins that regulate transcription by all three RNAP copurified with TFIIIB subunit Brf1 in *L. major* (Florencio-Martínez et al. 2021). Of note, immunoprecipitation assays led to the identification of chromatin modifiers and regulators of RNAP II and I transcription as putative interactors of Tau91 in yeast (Bhalla et al. 2019b). Thus, it is possible that TFIIIC and TFIIIB participate in a crosstalk between the transcription machineries of all three nuclear RNAP to regulate global transcription.

Regarding transcription of protein-coding genes, one subunit of the PAF1 complex was identified with LmTau95 (Table 2). This complex is implicated in the control of RNAP II and RNAP I gene expression at multiple steps, including transcriptional elongation and termination, RNA processing, and export (Francette et al. 2021). Interestingly, it was recently shown that yeast PAF1 also interacts with RNAP III transcriptional complexes to repress expression of tRNA genes (Bhalla et al. 2019a). In *T. brucei*, the CTR9 subunit of PAF1 is an essential protein whose knock-down produces gene expression defects (Ouna et al. 2012). With both parasites we also identified RuvB-like DNA helicases, which are part of large multiprotein complexes, such as NuA4 and INO80, involved in chromatin remodeling and transcription (Ohdate et al. 2003). Also, TFIIF-stimulated CTD phosphatase, and retrotransposon hot spot protein 5 copurified with TbTau95. Interestingly, retrotransposon hot spot proteins are trypanosome-specific factors that interact with RNAP II (Das et al. 2006), and their depletion impairs mRNA synthesis (Florini et al. 2019). Interaction of TFIIIC with proteins that regulate RNAP II transcription, such as N-MYC and Aurora-A, was found in neuroblastoma cell lines, where TFIIIC is required for N-MYC-dependent promoter escape and pause release of RNAP II (Büchel et al. 2017).

Besides its role as a general transcription factor, in some organisms TFIIIC is involved in chromatin domain organization by binding to thousands of sequences known as extra TFIIIC sites, which are independent of RNAP III transcription (Donze 2012). Extra TFIIIC sites are commonly localized at the border of topological domains, where TFIIIC interacts with cohesin and condensin complexes (Van Bortle et al. 2014; Yuen et al. 2017). Interestingly, among the proteins that copurified with TbTau95-PTP, although with a coverage of less than 20%, we found subunits SMC1 (Tb927.9.11850, average coverage of 10%) and SMC3 (Tb927.5.3510, 5% coverage) of the cohesin complex, as well as a putative ortholog of the cohesin-associated protein Pds5 (HHpred probability of 100%, E-value 2.9 × 10^−102^) (Tb927.11.6420, 4.5% coverage) (Supplemental Table S1). Moreover, we identified the SMC2 subunit (Tb927.10.10340, 4% coverage) of the condensin complex. Thus, it is possible that TFIIIC participates in three-dimensional chromosome organization in *T. brucei*, as reported in other species. Future ChIP-seq studies with TbTau95 will help to establish if extra TFIIIC sites are indeed present in the *T. brucei* genome. It is worth noting that no cohesin or condensin subunits copurified with LmTau95.

Factors involved in DNA replication were also found (Tables 1 and 2), implying that transcription and DNA replication are coupled in trypanosomatid parasites, as described in other organisms (Chen et al. 2019). In our experiments, actin was copurified with TbTau95 and LmTau95. Even though it is an abundant protein that is considered a regular contaminant in affinity purification assays, it has been shown that actin stably associates with RNAP III and is required for transcription of human U6 snRNA in a reconstituted in vitro transcription system (Hu et al. 2004). Thus, we cannot rule out the possibility that actin interacts with Tau95 in trypanosomatids to regulate RNAP III transcription.

In conclusion, in this work, we show that, contrary to the established assumption, a TFIIIC complex is indeed present in the trypanosomatid parasites *T. brucei* and *L. major*. Tandem affinity purifications using TbTau95-PTP and LmTau95-PTP as molecular baits allowed the identification of subunits Tau55 and Tau131, which together with Tau95 constitute the τA subcomplex of TFIIIC. Tau138 was the only subunit of the τB subcomplex that we identified. Although we cannot rule out the presence of novel TFIIIC subunits in trypanosomatids, our data strongly suggest that only four subunits integrate TFIIIC in *T. brucei* and *L. major*. Several other factors copurified with the tagged proteins, including several RNAP subunits and regulators of transcription by RNAPs III and II. Thus, it is feasible that TFIIIC is involved in the control and coordination of global transcription in trypanosomatids. Also, the association of TbTau95 with cohesin and condensing subunits supports the role of TFIIIC in chromatin domain organization in trypanosomatids, as reported in other organisms.

## Supplementary Information

Below is the link to the electronic supplementary material.

Supplementary file1 (PDF 819 KB)

Supplementary file2 (XLSX 6148 KB)

## Data Availability

The mass spectrometry proteomics data have been deposited to the ProteomeXchange Consortium (http://www.proteomexchange.org/) via the PRIDE partner repository (Perez-Riverol et al. 2022) with the dataset identifier PXD045036 and 10.6019/PXD045036.
