# Supplementary material for: Characterization of Tau95 led to the identification of a four-subunit TFIIIC complex in trypanosomatid parasites
Source: Appl Microbiol Biotechnol. 2024 Jan 10;108:109. doi: 10.1007/s00253-023-12903-8 (PMC10781861; doi:10.1007/s00253-023-12903-8)
Supplement: Supplementary file 1 [file 253_2023_12903_MOESM1_ESM.pdf]

Supplemental Figures

Applied Microbiology and Biotechnology

Characterization of Tau95 led to the identification of a four-subunit TFIIIC complex in trypanosomatid parasites

Fabiola Mondragón-Rosas[1], Luis E. Florencio-Martínez[1], Gino S. Villa-Delavequia[1], Rebeca G. Manning-Cela[2], Julio C. Carrero[3], Tomás Nepomuceno-Mejía[1], Santiago Martínez-Calvillo[1,*]

Supplemental Figure S1

a

	**Tb** 647aa	**Lm** 700aa	**Sp** 456aa	**Sc** 649aa	**Hs** 519aa	**Mm** 520aa	**Rn** 515aa	**At** 554	**Ce** 433	**Pf** 916aa	**Tg** 637aa	**Nf** 731aa
**Tb**	100	34.92	17.09	14.37	15.47	14.33	13.97	16.85	14.24	14.92	17.53	11.70
**Lm**		100	16.78	14.40	14.87	15.41	14.45	16.38	15.59	15.24	19.77	14.25
**Sp**			100	24.05	26.58	27.00	27.00	19.15	24.44	22.74	19.65	22.82
**Sc**				100	17.86	17.86	18.21	21.07	19.33	21.26	20.65	16.18
**Hs**					100	85.36	83.88	25.51	27.51	20.92	21.98	27.01
**Mm**						100	95.15	24.55	27.99	20.68	20.99	27.63
**Rn**							100	23.68	28.23	20.68	20.99	27.63
**At**								100	20.16	20.45	20.22	21.98
**Ce**									100	21.95	19.30	23.66
**Pf**										100	21.48	20.16
**Tg**											100	21.40
**Nf**												100

b

	**Tb** 647 aa	**Tev** 647 aa	**Tco** 603 aa	**Tc** 597 aa	**Tv** 587 aa	**Lmx** 693 aa	**Lbr** 693 aa	**Lin** 698 aa	**Lta** 719 aa	**Ld** 697 aa	**Lm** 700 aa
**Tb**	100.00	99.54	54.85	47.95	48.23	34.72	34.33	34.39	32.69	34.44	33.83
**Tev**		100.00	55.02	48.12	48.23	35.06	34.67	34.72	33.01	34.78	34.00
**Tco**			100.00	46.06	45.94	34.31	34.36	33.45	33.96	33.50	32.71
**Tc**				100.00	50.09	36.78	36.95	36.52	35.66	36.59	35.94
**Tv**					100.00	34.29	34.72	33.87	32.14	33.93	33.15
**Lmx**						100.00	83.53	93.36	85.09	93.50	92.64
**Lbr**							100.00	84.49	80.15	84.33	81.77
**Lin**								100.00	84.13	99.57	92.84
**Lta**									100.00	84.26	83.14
**Ld**										100.00	92.97
**Lm**											100.00

Supplemental Fig. S1. Percent identities among amino acid sequences of Tau95 from different groups of organisms (a) and trypanosomatids (b). The species analyzed are: (a) *Trypanosoma brucei* (Tb, Tb927.10.980), *Leishmania major* (Lm, LmjF.21.1100), *Schizosaccharomyces pombe* (Sp, NP_593297), *Saccharomyces cerevisiae* (Sc, GFP66923), *Homo sapiens* (Hs, AAH11355), *Mus musculus* (Mm, NP_001277413), *Rattus norvegicus* (Rn, NP_001073410), *Arabidopsis thaliana* (At, NP_197833), *Caenorhabditis elegans* (Ce, CAA84675), *Plasmodium falciparum* (Pf, KNG75525.1), *Toxoplasma gondii* (Tg, ESS35509), and *Naegleria fowleri* (Nf, KAF0983925); (b) *T. brucei* (Tb, Tb927.10.980), *Trypanosoma evansi* (Tev, STIB805.10.1070), *Trypanosoma congolense* (Tco, TcIL3000_10_840), *Trypanosoma cruzi* (Tc, TcCLB.511131.20), *Trypanosoma vivax* (Tv, TvY486_1000950), *Leishmania mexicana* (Lmx, LmxM.21.1100), *Leishmania braziliensis* (Lbr, LbrM.30.3810), *Leishmania infantum* (Lin, LinJ.21.1340), *Leishmania tarentolae* (Lta, LtaP21.1280), *Leishmania donovani* (Ld, LdBPK_211340.1), and *L. major* (Lm, LmjF.21.1100).

Supplemental Figure S2

```
T. brucei        558 GWIGS-(48) -ASSTSSVSPESYGDDDNEDSLESVSPVDDLSQHPDDG     647
L. major         609 GWLSE-(46) -ESSTSSLRDEELEDDEDEDDSAMFSVSAGDLSEAADDDDE 700
S. pombe         349 GWYRS-(82) -EHEGFEDLEEIDDDYDDIFGD                      456
S. cerevisiae    468 GWFKE-(122)-DDDVDDVDADEEEQE         -(40)-            649
H. sapiens       437 GWCLP-(41) -ESGEDEEDEEEEEEEEEDFKPSDGSENEMETEILDYV     519
```

Supplemental Fig. S2. Sequence alignment of acidic tail residues of Tau95 from *T. brucei*, *L. major*, *S. pombe*, *S. cerevisiae* and *H. sapiens*. Acidic residues are shown in red.

Supplemental Figure S3

Tb927.11.1590
Annotated as: Hypothetical protein
Putative ortholog of *S. pombe* **Tau55 (Sfc7)**. Probability: 82.97%. E-value: 1.8

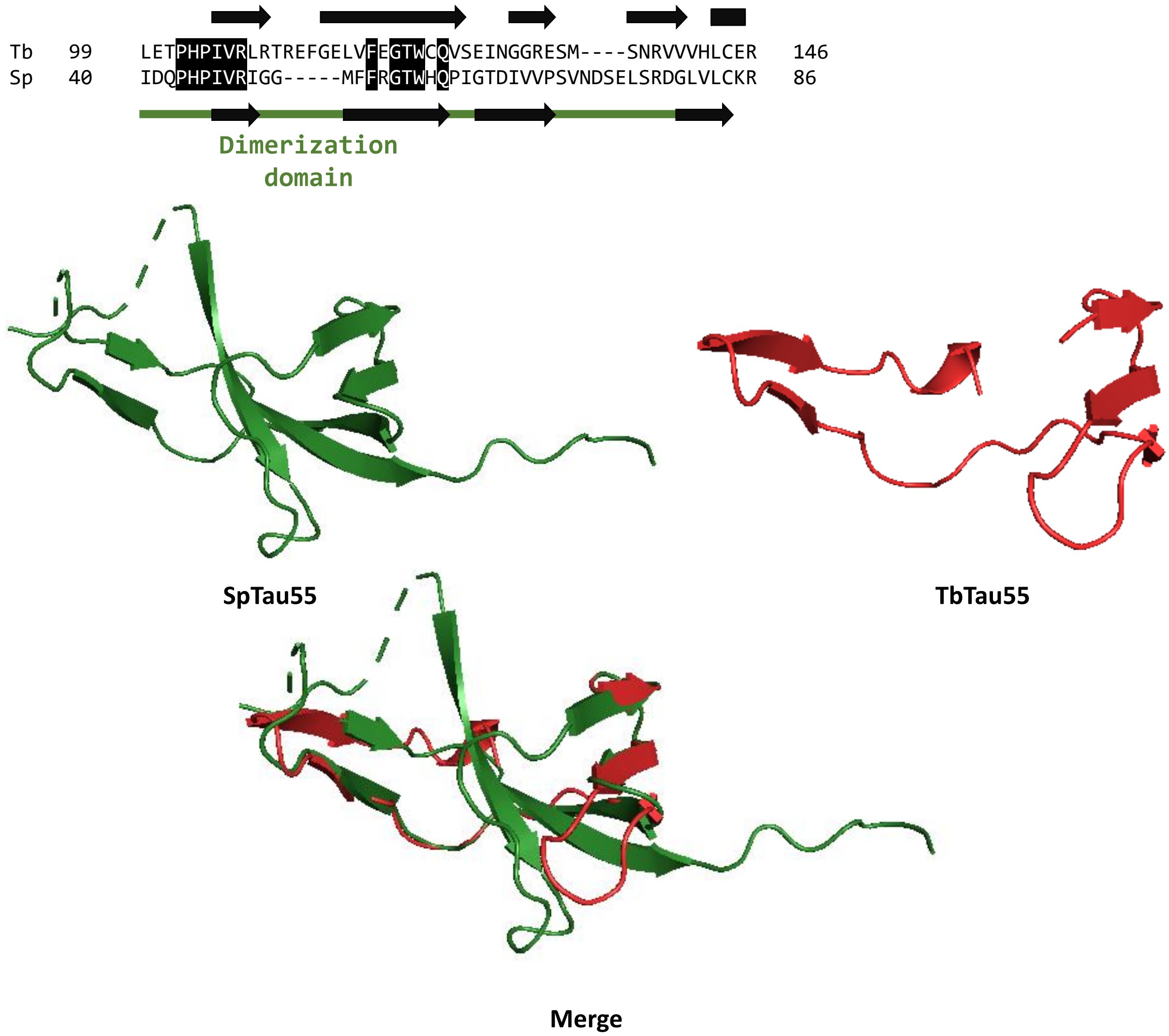

LmjF.27.0990

Annotated as: Hypothetical protein

Putative ortholog of *S. pombe* **Tau55 (Sfc7)**. Probability: 84.25%. E-value: 1.5

```
Lm   140   LETDAPRLLLNGGTPHEMLFEGQWTEVTG   168
Sp   39    IDQPHPIVRIGG-----MFFRGTWHQPIG   62
```

Dimerization domain

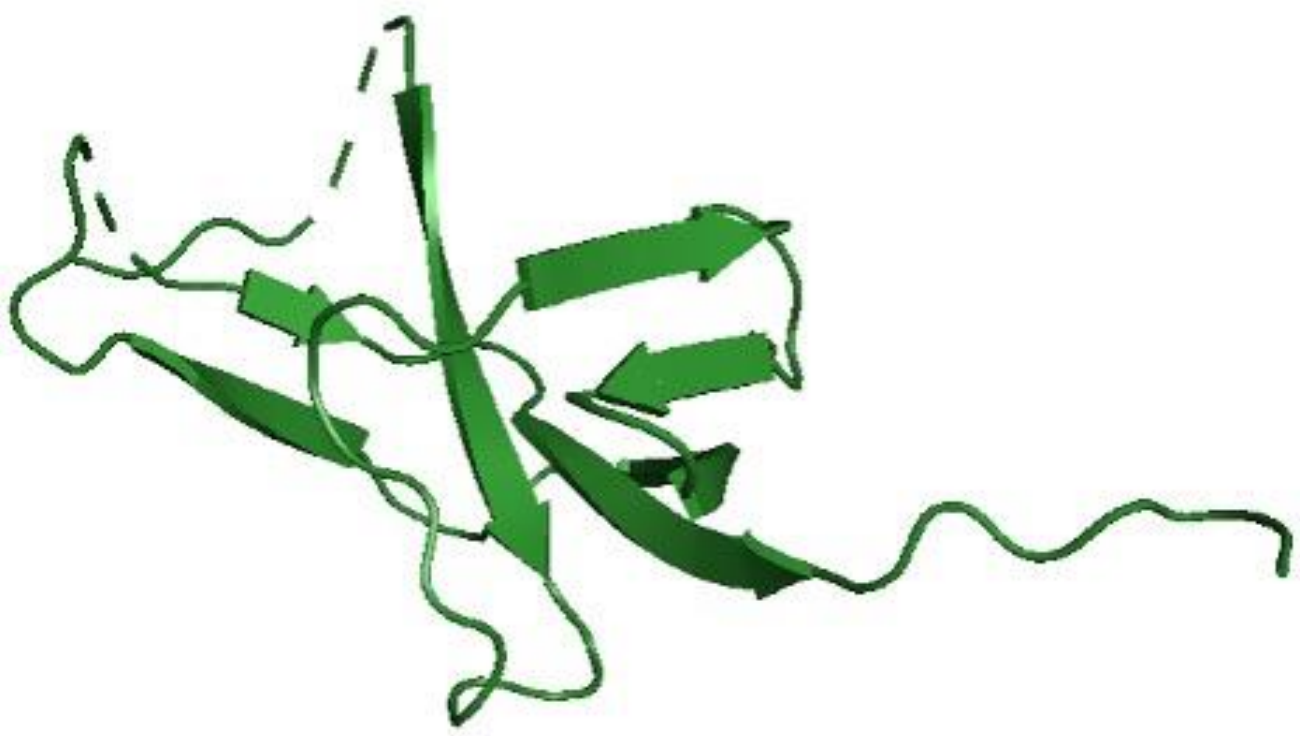

SpTau55

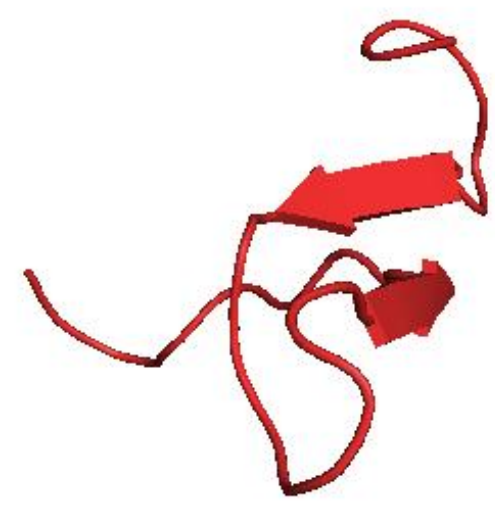

LmTau55

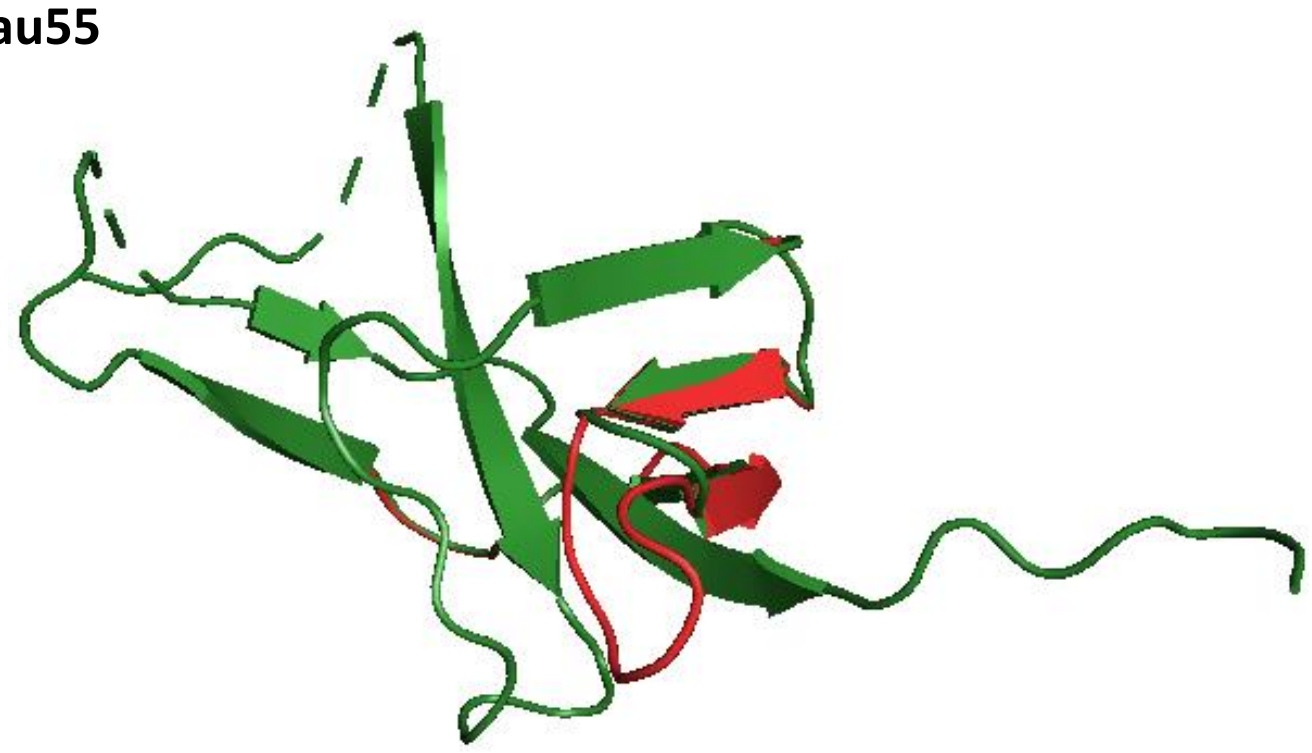

Merge

Tb927.1.3860

Annotated as: Hypothetical protein

Putative ortholog of *S. cerevisiae* **Tau131**. Probability: 100%. E-value: $3.7e^{-56}$

```
Tb   28   TAFSSMELHRRFEEHVAKAQQLFLQGNKDGAEQEATLALRIHPTDH-LFALLAVIAESKGQFNRASDLRLLQAFMA-NDA   105
Sc   117  RQRKERVLDPEVAQLLSQANEAFVRNDLQVAERLFNEVIKKDARNFAAYETLGDIYQLQGRLNDCCNSWFLAAHLNASDW   196
                        TPR 1                                     TPR 2

Tb   106  VLWEELLHEFMQERLYYKSAVCLHRLAVLETRDKVRYRTLQLQLADLYIGLGEFKRAAHILISLWNGSRCRDFEVFAMLS   185
Sc   197  EFWKIVAILSADLDHVRQAIYCFSRVISLNPME---WE-SIYRRSMLYKKTGQLARALDGFQRLYM-YNPYDANILRELA   271
                 TPR 3                                              TPR 4

Tb   186  SLFFQLGRWNSLHRLIESSLKSTFRPVVCTMEGGEKPPDAPSSRLGREPSTVEGKEELTAPMARRRVPR-RVRFFGVDDN   264
Sc   272  ILYVDYDRIEDSIELYMKVFNANVER-REA--------------ILAALENA---------LDSSDEESAAEGE-DADEK   326
                                   TPR 5

Tb   265  DECDNDTDSVVKRAEGIQQAGSLSQPTPTVDNTCLAEALCEGDDFDFTSEGCDSQSVHGIRSGACATPSAVIHSMYGDSV   344
Sc   327  -----EP-----------------------LEQ-------DEDRQ--MF-P--------------DI-NW-KKIDAK-Y   350

Tb   345  KFRTATDKKNFLTLVNVHAELLNEEGNFPD-TVRLVEFAAGCLN----------------------------------V   388
Sc   351  -----KCIPFDWSSLNILAELFLKLAVSEVDGIKTIKKCARWIQRRESQTFWDHVPDDSEFDNRRFKNSTFDSLLAAEKE   425
                                TPR 6

Tb   389  SLLELHPDLLVRLGVAYAFLGGMEQQCREVFHHLVDTCPMAEYGDVLYDAANALQQVGLHTEARMLFQTVRRYHEFRMKG   468
Sc   426  KSYNIPIDIRVRLGLLRLNTDNLVEA-LNHFQCLYDE-TFSDVADLYFEAATALTRAEKYKEAIDFFTPLLSLEEW----   499
                              TPR 7                                  TPR 8

Tb   469  SNGNADDGNNLDDEEKTEVKTVFVAALFAESQCEASLGNIEVACDNLCRVLEVDPHHLQSRLALGRLCMYDMNDTERAIE   548
Sc   500  ---------------------RTTDVFKPLARCYKEIESYETAKEFYELAIKSEPDDLDIRVSLAEVYY-RLNDPETFKH   557
                                       TPR 9                              TPR 10

Tb   549  VLTPRESEP    557
Sc   558  MLVDVVEMR    566
```

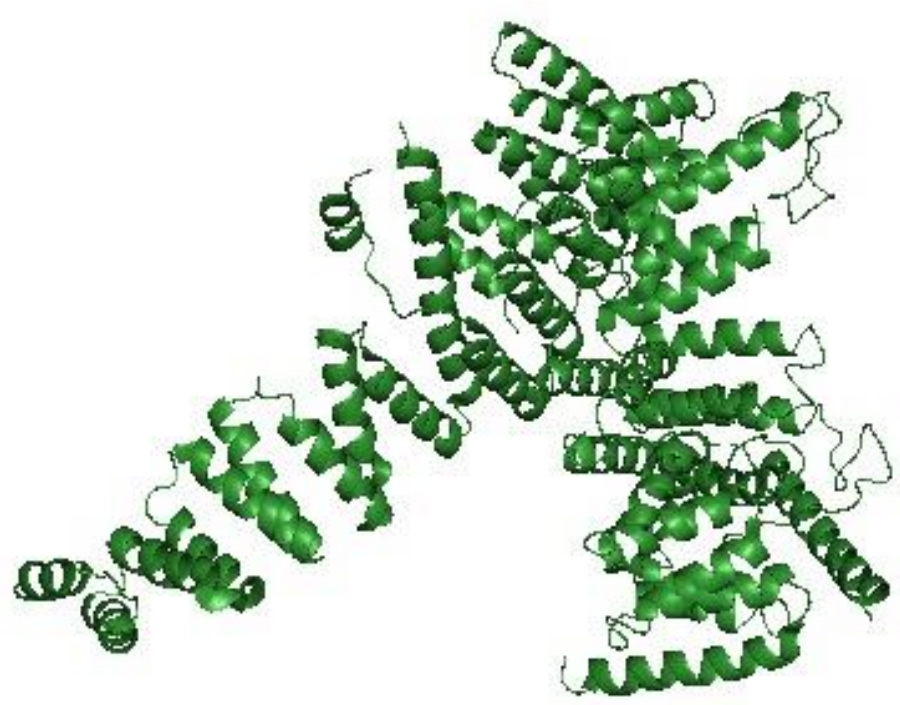

ScTau131

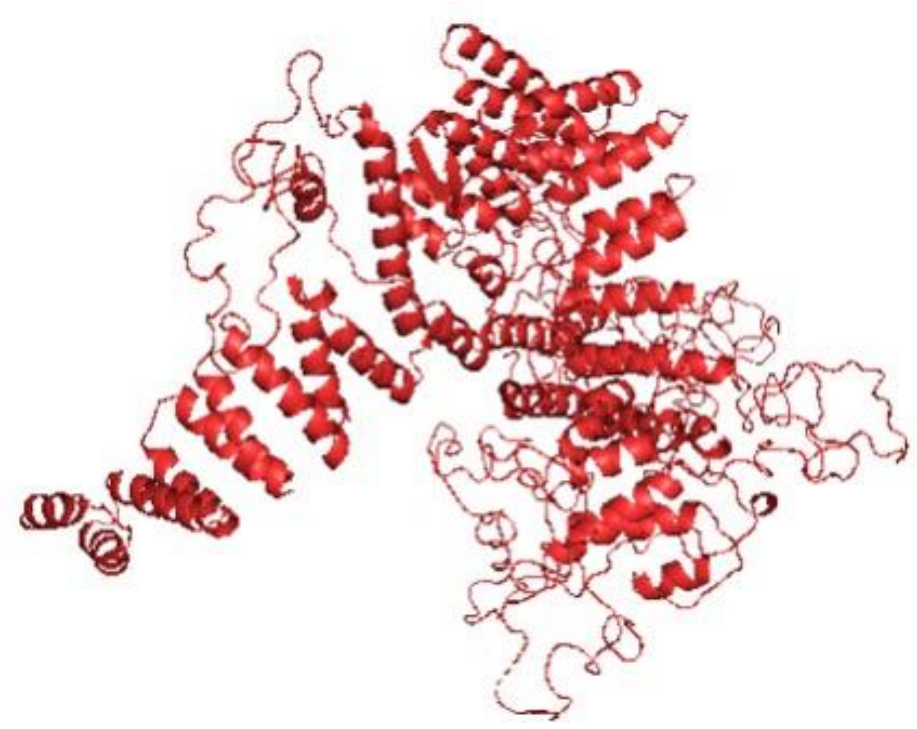

TbTau131

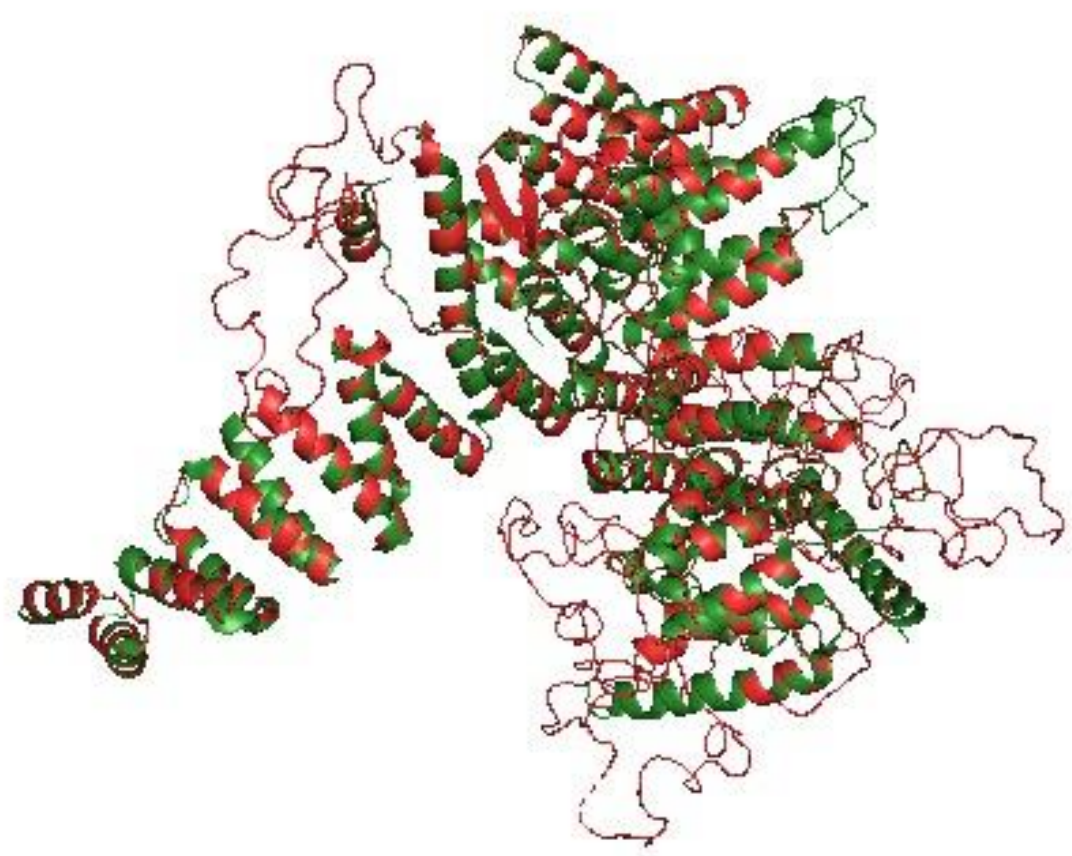

Merge

LmjF.12.0560

Annotated as: Hypothetical protein

Putative ortholog of *S. cerevisiae* **Tau131**. Probability: 100%. E-value: $1.3e^{-37}$

```
Lm    33    SSSALHSTFEAHVTAAQNSFLRGDSQAAEHEAVQALRIRPTAH-LFALLAVIAESKGKFDQASDFRLLQAFLA-RDIVLW    110
Sc    120   KERVLDPEVAQLLSQANEAFVRNDLQVAERLFNEVIKKDARNFAAYETLGDIYQLQGRLNDCCNSWFLAAHLNASDWEFW    199
                         TPR 1                                     TPR 2

Lm    111   EELLHDFMTQQLYYKSVVCLQRMSALEKNQERYRALQLQLADLFIGLGEIRRASNVLVPLWNSSRCRDFEVFALLSSLYF    190
Sc    200   KIVAILSADLDHVRQAIYCFSRVISLN--PMEWE-SIYRRSMLYKKTGQLARALDGFQRLYM-YNPYDANILRELAILYV    275
                     TPR 3                                 TPR 4

Lm    191   QLGKWASLQRLIESSVKHAFQLLPTAEVTGATTTAGETMGHGAEATAQVSSSTPGTEQQLSSPPPDSSISEQRVSKRVRF    270
Sc    276   DYDRIEDSIELYMKVFNANVERRE---------AILAA-----------LENALDSS------DEE--------------    315
                                    TPR 5

Lm    271   FGMDAGEDGAAAPVTADLTAPTAPMSLSPAEASLDEFRFDDFISPASPLSHPPSSLRPGDTASSTGAHRGGVVHQLYGER    350
Sc    316   -------------------------S-AA-EGEDADEK------E--P--------------------------------    328

Lm    351   IELRSSQDQKNFLTLVNVHAELLNEQGNFSDTVQ-LLRMAAGCLDVDLIELPPDLLVRLGVAYAFLGGVYEQPCRD-VFQ    428
Sc    329   ----------------------LEQDEDRQMFPDINWKKIDAKY--KCIPFDWSSLNILAELFLKLAV-----SEVDGIK    379
                                                                                TPR 6

Lm    429   HLVAH-C----------------------------------------SMEDYGDVLLDAAMSLQKVSMHAEAAQVYVTLA    467
Sc    380   TIKKCARWIQRRESQTFWDHVPDDSEFDNRRFKNSTFDSLLAAEKEKSYNIPIDIRVRLGLLRLNTDNLVEALNHFQCLY    459
                                                                                TPR 7

Lm    468   RYHQFVYDRVRRQVGVAEAELTLMQDTAPLDQVQAATDALKALKTEEADLKTVLAAALQGQAQSAYARELYEEAKEYAEC    547
Sc    460   DETFS-----------------------------------------------DVADLYFEAATALTRAEKYKEAIDFFTP    492
                                                                                TPR 8

Lm    548   VLQLEPT-HLQARLLLGRYYFYQRKDTAAAVEVLTPSKHEPALQRIQLGVFLVHVFQRAKRYVEAIALGVSIFELLLSSE    626
Sc    493   LLSLEEWRTTDVFKPLARCYK-EIESYETAKEFYELAIKSEPDD-LDIRVSLAEVYYRLNDPETFKHMLVDVVEMRKHQV    570
                        TPR 9                                TPR 10
```

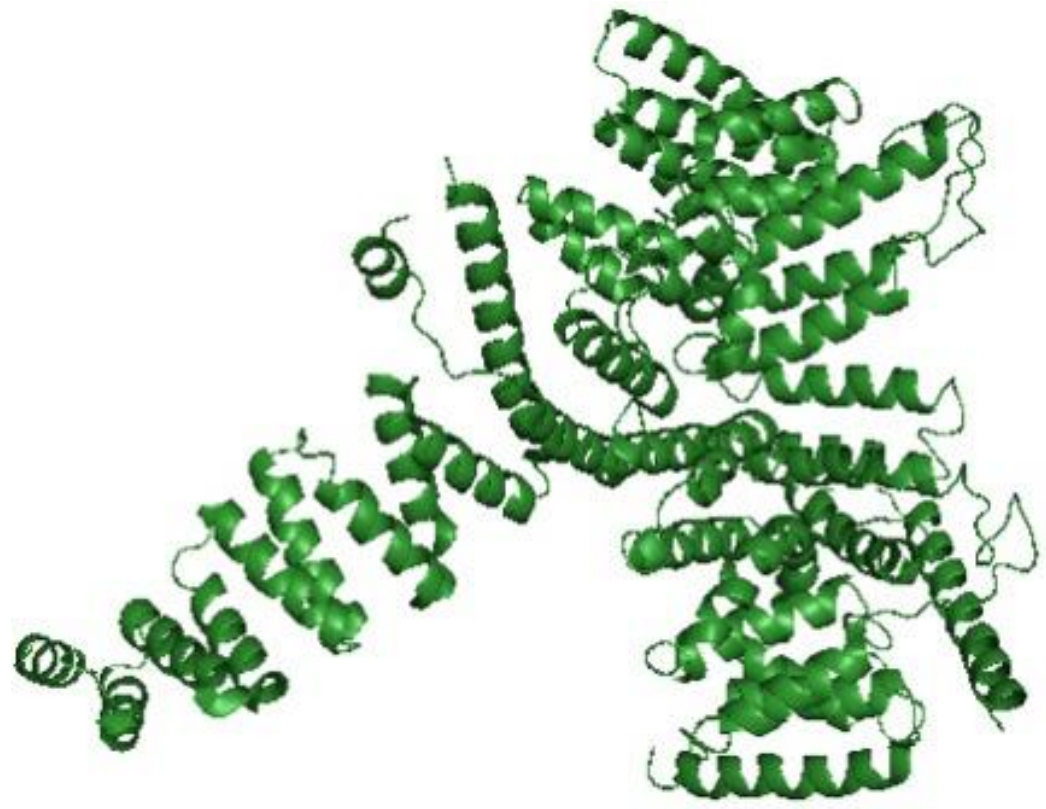

ScTau131

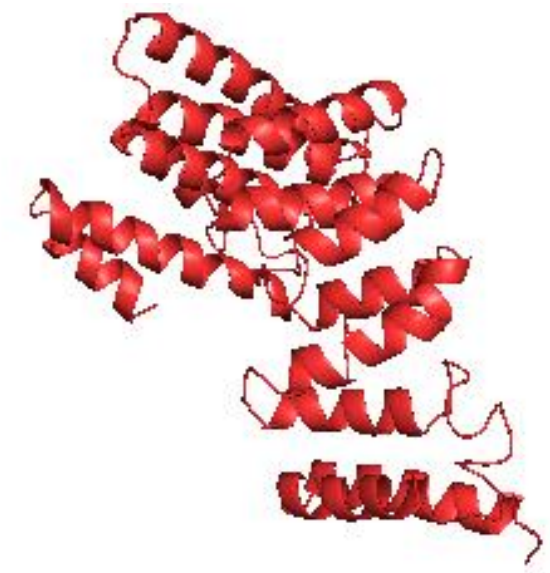

LmTau131

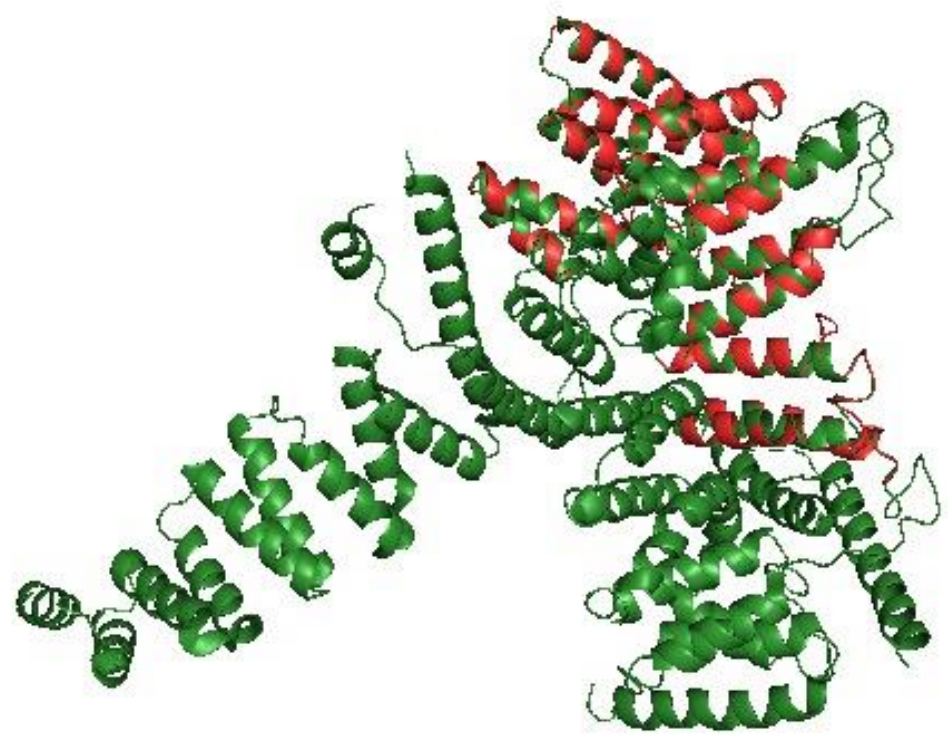

Merge

Tb927.11.4520

Annotated as: Hypothetical protein

Putative ortholog of *S. cerevisiae* **Tau138**. Probability: 78.91%. E-value: 13

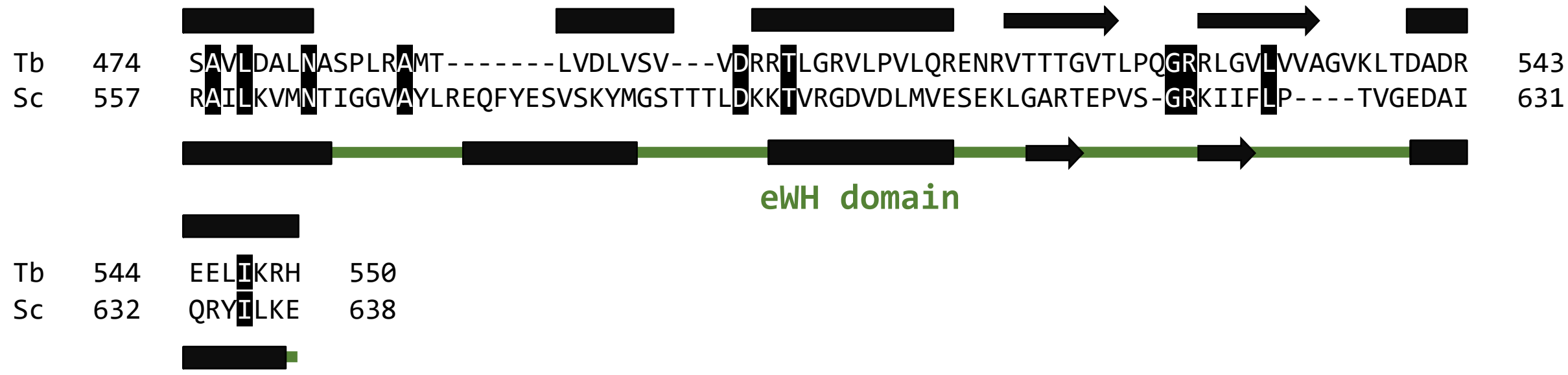

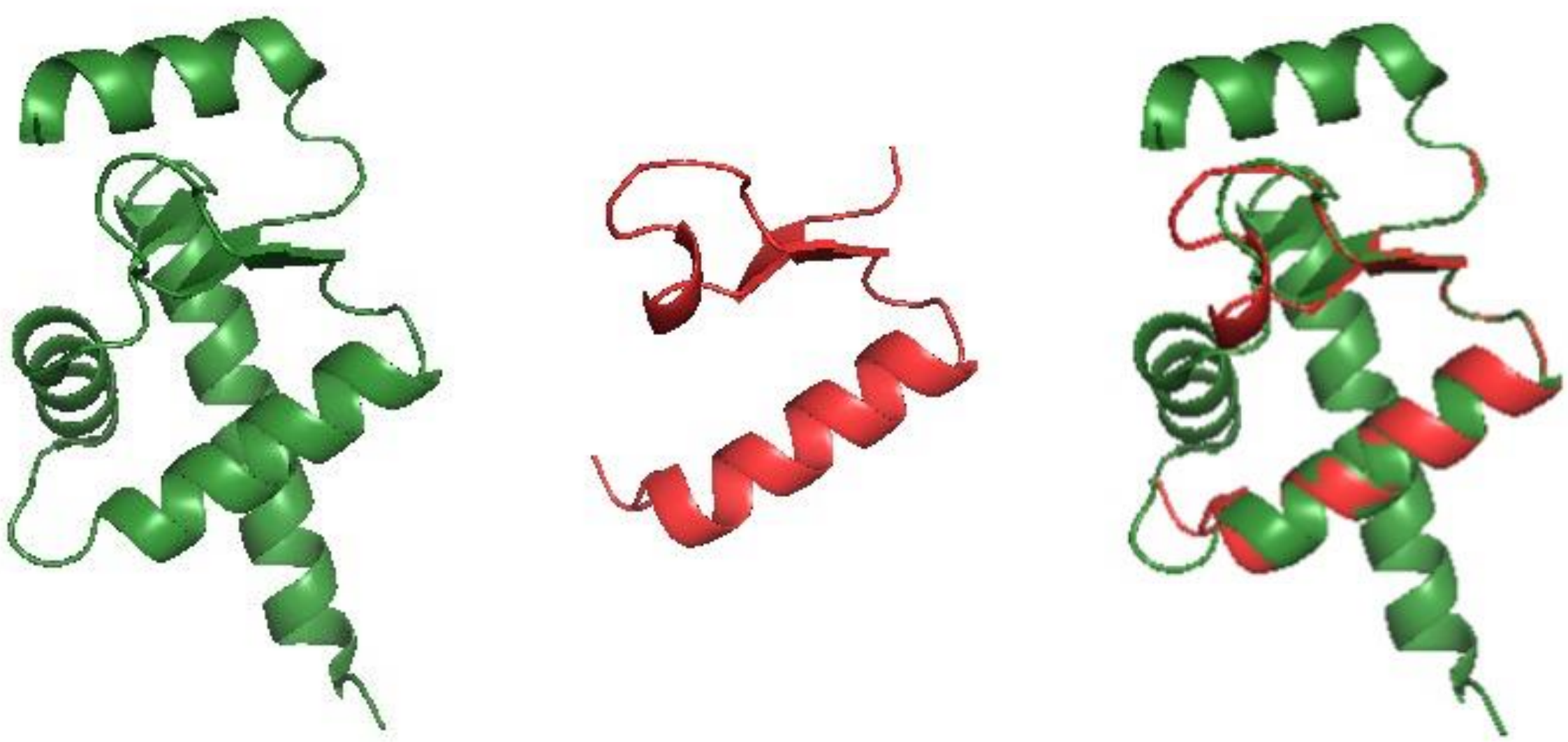

ScTau138 **TbTau138** **Merge**

LmjF.13.0270

Annotated as: Hypothetical protein

Putative ortholog of *S. cerevisiae* **Tau138**. Probability: 30.23%. E-value: 240

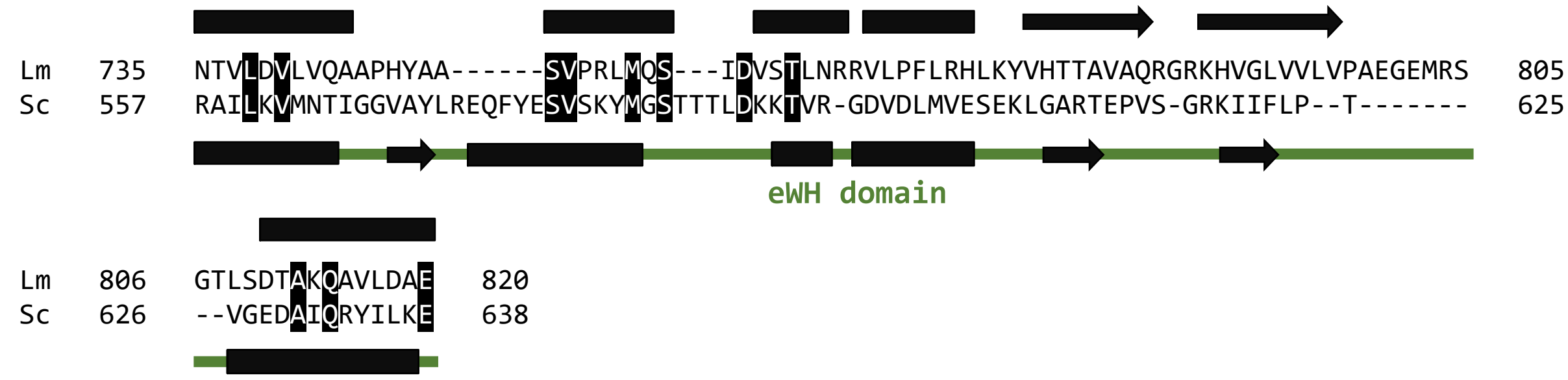

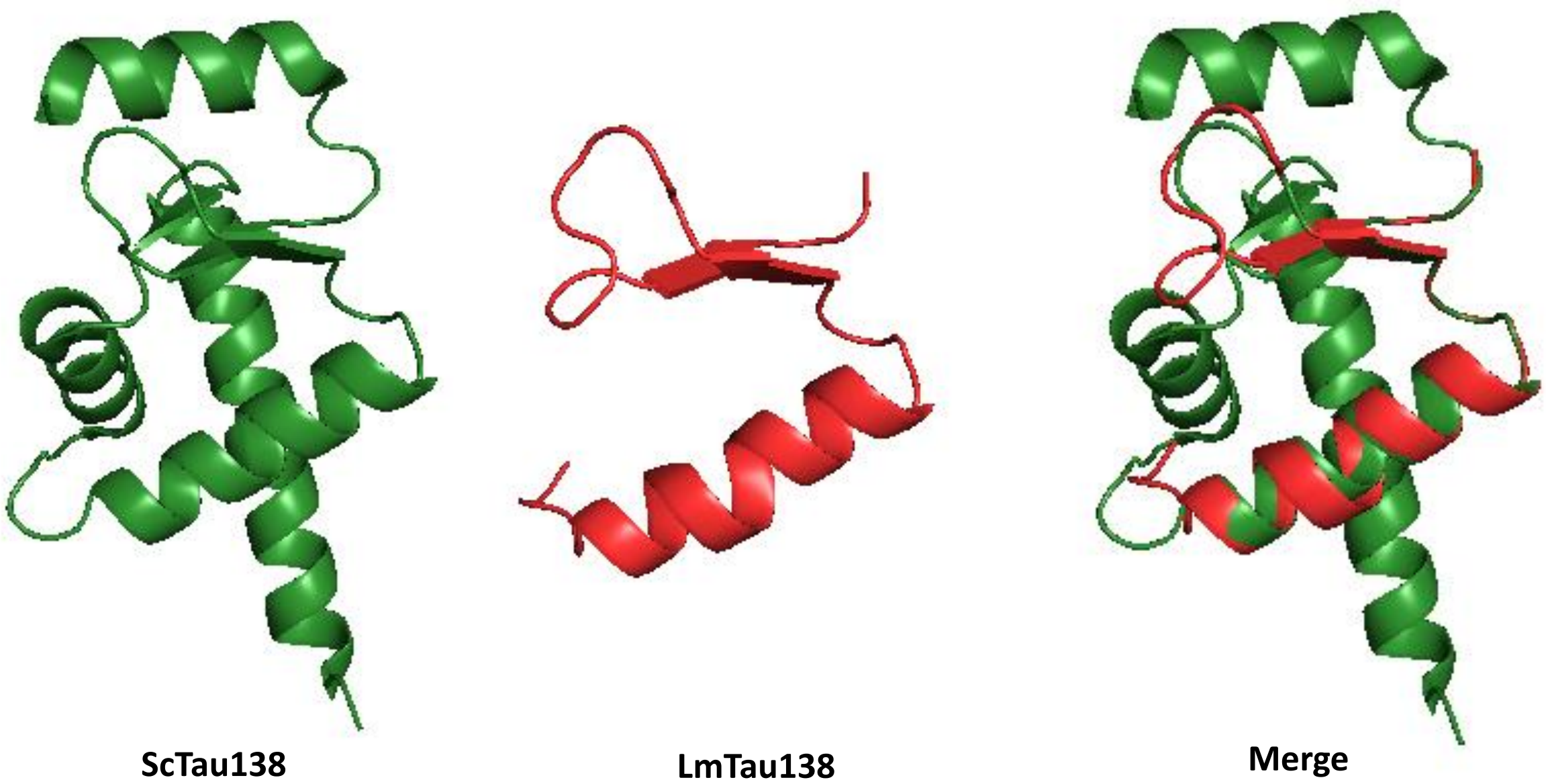

Supplemental Fig. S3. Sequence and structure analyses of the presumed orthologs of Tau55, Tau131 and Tau138 in *T. brucei* and *L. major*. The HHpred (https://toolkit.tuebingen.mpg.de/tools/hhpred) and Swiss-Model (http://swissmodel.expasy.org/interactive) programs were used to analyze the sequence and predicted three-dimensional structure of the three proteins. Conserved domains are indicated in the sequence alignments. For Tau131, the alignment shown corresponds to the first 10 TPRs (in the N-terminal region of the protein); the C-terminal region is not presented.
