# Supplementary material for: Characterization of Tau95 led to the identification of a four-subunit TFIIIC complex in trypanosomatid parasites
Source: Appl Microbiol Biotechnol. 2024 Jan 10;108(1):109. doi: 10.1007/s00253-023-12903-8 (PMC10781861; doi:10.1007/s00253-023-12903-8)
Supplement: Supplementary file 1 — Supplementary file1 (PDF 819 KB) [file 253_2023_12903_MOESM1_ESM.pdf]

Supplemental Figures

Applied Microbiology and Biotechnology

Characterization of Tau95 led to the identification of a four-subunit TFIIIC complex in trypanosomatid parasites

Fabiola Mondragón-Rosas[1], Luis E. Florencio-Martínez[1], Gino S. Villa-Delavequia[1], Rebeca G. Manning-Cela[2], Julio C. Carrero[3], Tomás Nepomuceno-Mejía[1], Santiago Martínez-Calvillo[1,*]

Supplemental Figure S1

a

	Tb 647aa	Lm 700aa	Sp 456aa	Sc 649aa	Hs 519aa	Mm 520aa	Rn 515aa	At 554	Ce 433	Pf 916aa	Tg 637aa	Nf 731aa
**Tb**	100	34.92	17.09	14.37	15.47	14.33	13.97	16.85	14.24	14.92	17.53	11.70
**Lm**		100	16.78	14.40	14.87	15.41	14.45	16.38	15.59	15.24	19.77	14.25
**Sp**			100	24.05	26.58	27.00	27.00	19.15	24.44	22.74	19.65	22.82
**Sc**				100	17.86	17.86	18.21	21.07	19.33	21.26	20.65	16.18
**Hs**					100	85.36	83.88	25.51	27.51	20.92	21.98	27.01
**Mm**						100	95.15	24.55	27.99	20.68	20.99	27.63
**Rn**							100	23.68	28.23	20.68	20.99	27.63
**At**								100	20.16	20.45	20.22	21.98
**Ce**									100	21.95	19.30	23.66
**Pf**										100	21.48	20.16
**Tg**											100	21.40
**Nf**												100

b

	Tb 647 aa	Tev 647 aa	Tco 603 aa	Tc 597 aa	Tv 587 aa	Lmx 693 aa	Lbr 693 aa	Lin 698 aa	Lta 719 aa	Ld 697 aa	Lm 700 aa
**Tb**	100.00	99.54	54.85	47.95	48.23	34.72	34.33	34.39	32.69	34.44	33.83
**Tev**		100.00	55.02	48.12	48.23	35.06	34.67	34.72	33.01	34.78	34.00
**Tco**			100.00	46.06	45.94	34.31	34.36	33.45	33.96	33.50	32.71
**Tc**				100.00	50.09	36.78	36.95	36.52	35.66	36.59	35.94
**Tv**					100.00	34.29	34.72	33.87	32.14	33.93	33.15
**Lmx**						100.00	83.53	93.36	85.09	93.50	92.64
**Lbr**							100.00	84.49	80.15	84.33	81.77
**Lin**								100.00	84.13	99.57	92.84
**Lta**									100.00	84.26	83.14
**Ld**										100.00	92.97
**Lm**											100.00

Supplemental Fig. S1. Percent identities among amino acid sequences of Tau95 from different groups of organisms (a) and trypanosomatids (b). The species analyzed are: (a) *Trypanosoma brucei* (Tb, Tb927.10.980), *Leishmania major* (Lm, LmjF.21.1100), *Schizosaccharomyces pombe* (Sp, NP_593297), *Saccharomyces cerevisiae* (Sc, GFP66923), *Homo sapiens* (Hs, AAH11355), *Mus musculus* (Mm, NP_001277413), *Rattus norvegicus* (Rn, NP_001073410), *Arabidopsis thaliana* (At, NP_197833), *Caenorhabditis elegans* (Ce, CAA84675), *Plasmodium falciparum* (Pf, KNG75525.1), *Toxoplasma gondii* (Tg, ESS35509), and *Naegleria fowleri* (Nf, KAF0983925); (b) *T. brucei* (Tb, Tb927.10.980), *Trypanosoma evansi* (Tev, STIB805.10.1070), *Trypanosoma congolense* (Tco, TcIL3000_10_840), *Trypanosoma cruzi* (Tc, TcCLB.511131.20), *Trypanosoma vivax* (Tv, TvY486_1000950), *Leishmania mexicana* (Lmx, LmxM.21.1100), *Leishmania braziliensis* (Lbr, LbrM.30.3810), *Leishmania infantum* (Lin, LinJ.21.1340), *Leishmania tarentolae* (Lta, LtaP21.1280), *Leishmania donovani* (Ld, LdBPK_211340.1), and *L. major* (Lm, LmjF.21.1100).

Supplemental Figure S2

```
T. brucei         558 GWIGS-(48) -ASSTSSVSPESYGDDDNEDSLESVSPVDDLSQHPDDG     647
L. major          609 GWLSE-(46) -ESSTSSLRDEELEDDEDEDDSAMFSVSAGDLSEAADDDDE  700
S. pombe          349 GWYRS-(82) -EHEGFEDLEEIDDDYDDIFGD                      456
S. cerevisiae     468 GWFKE-(122)-DDDVDDVDADEEEQE           -(40)-          649
H. sapiens        437 GWCLP-(41) -ESGEDEEDEEEEEEEEEEDFKPSDGSENEMETEILDYV     519
```

Supplemental Fig. S2. Sequence alignment of acidic tail residues of Tau95 from *T. brucei*, *L. major*, *S. pombe*, *S. cerevisiae* and *H. sapiens*. Acidic residues are shown in red.

Supplemental Figure S3

Tb927.11.1590
Annotated as: Hypothetical protein
Putative ortholog of *S. pombe* **Tau55 (Sfc7)**. Probability: 82.97%. E-value: 1.8

```
Tb   99    LETPHPIVRLRTREFGELVFEGTWCQVSEINGGRESM----SNRVVVHLCER   146
Sp   40    IDQPHPIVRIGG-----MFFRGTWHQPIGTDIVVPSVNDSELSRDGLVLCKR   86
```

Dimerization domain

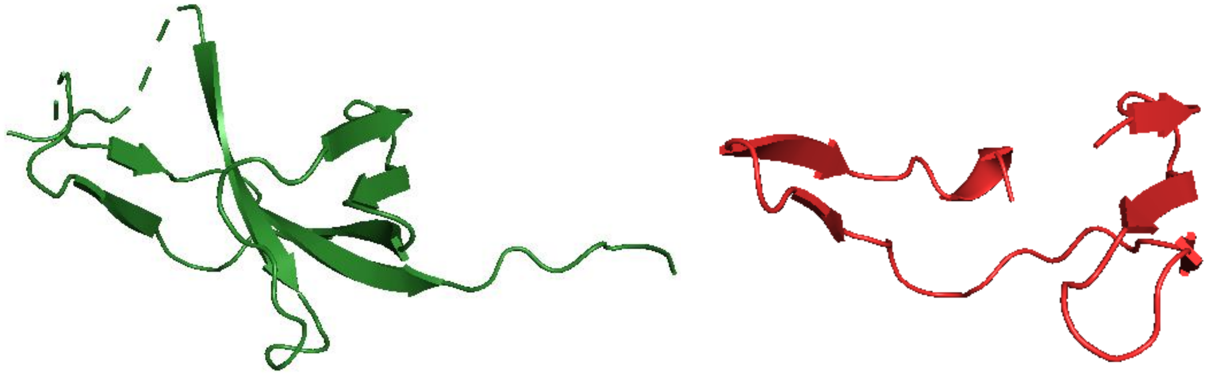

SpTau55 **TbTau55**

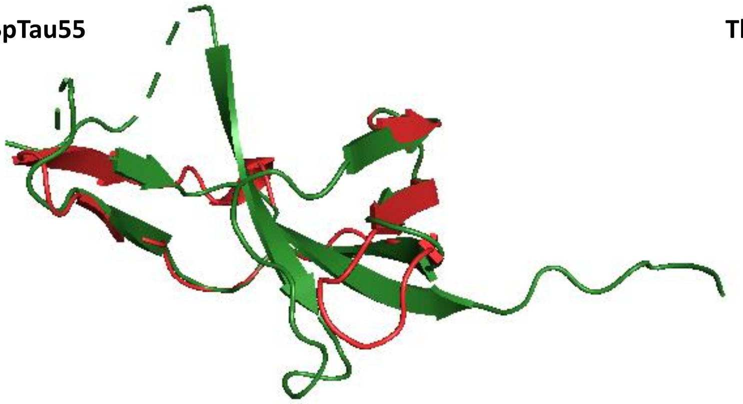

Merge

LmjF.27.0990

Annotated as: Hypothetical protein

Putative ortholog of *S. pombe* **Tau55 (Sfc7)**. Probability: 84.25%. E-value: 1.5

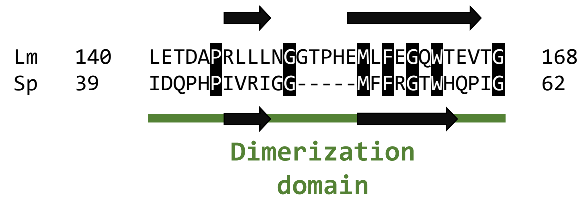

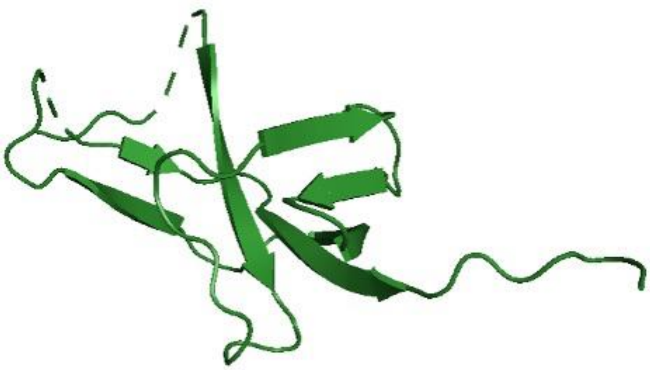

SpTau55

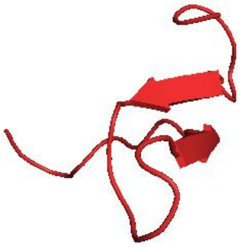

LmTau55

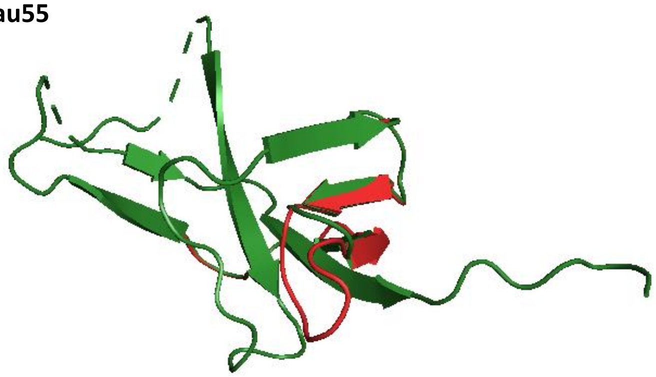

Merge

Tb927.1.3860

Annotated as: Hypothetical protein

Putative ortholog of *S. cerevisiae* **Tau131**. Probability: 100%. E-value: 3.7e-^{56}

```
Tb   28   TAFSSMELHRRFEEHVAKAQQLFLQGNKDGAEQEATLALRIHPTDH-LFALLAVIAESKGQFNRASDLRLLQAFMA-NDA   105
Sc   117  RQRKERVLDPEVAQLLSQANEAFVRNDLQVAERLFNEVIKKDARNFAAYETLGDIYQLQGRLNDCCNSWFLAAHLNASDW   196
                            TPR 1                               TPR 2

Tb   106  VLWEELLHEFMQERLYYKSAVCLHRLAVLETRDKVRYRTLQLQLADLYIGLGEFKRAAHILISLWNGSRCRDFEVFAMLS   185
Sc   197  EFWKIVAILSADLDHVRQAIYCFSRVISLNPME---WE-SIYRRSMLYKKTGQLARALDGFQRLYM-YNPYDANILRELA   271
                      TPR 3                                 TPR 4

Tb   186  SLFFQLGRWNSLHRLIESSLKSTFRPVVCTMEGGEKPPDAPSSRLGREPSTVEGKEELTAPMARRRVPR-RVRFFGVDDN   264
Sc   272  ILYVDYDRIEDSIELYMKVFNANVER-REA--------------ILAALENA---------LDSSDEESAAEGE-DADEK   326
                                       TPR 5

Tb   265  DECDNDTDSVVKRAEGIQQAGSLSQPTPTVDNTCLAEALCEGDDFDFTSEGCDSQSVHGIRSGACATPSAVIHSMYGDSV   344
Sc   327  -----EP------------------------LEQ-------DEDRQ--MF-P--------------DI-NW-KKIDAK-Y   350

Tb   345  KFRTATDKKNFLTLVNVHAELLNEEGNFPD-TVRLVEFAAGCLN-----------------------------------V   388
Sc   351  -----KCIPFDWSSLNILAELFLKLAVSEVDGIKTIKKCARWIQRRESQTFWDHVPDDSEFDNRRFKNSTFDSLLAAEKE   425
                                  TPR 6

Tb   389  SLLELHPDLLVRLGVAYAFLGGMEQQCREVFHHLVDTCPMAEYGDVLYDAANALQQVGLHTEARMLFQTVRRYHEFRMKG   468
Sc   426  KSYNIPIDIRVRLGLLRLNTDNLVEA-LNHFQCLYDE-TFSDVADLYFEAATALTRAEKYKEAIDFFTPLLSLEEW----   499
                                TPR 7                             TPR 8

Tb   469  SNGNADDGNNLDDEEKTEVKTVFVAALFAESQCEASLGNIEVACDNLCRVLEVDPHHLQSRLALGRLCMYDMNDTERAIE   548
Sc   500  ---------------------RTTDVFKPLARCYKEIESYETAKEFYELAIKSEPDDLDIRVSLAEVYY-RLNDPETFKH   557
                                               TPR 9                           TPR 10

Tb   549  VLTPRESEP    557
Sc   558  MLVDVVEMR    566
```

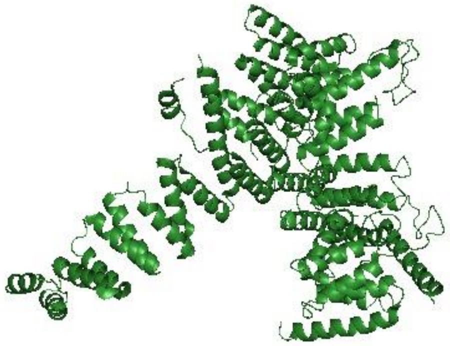

ScTau131

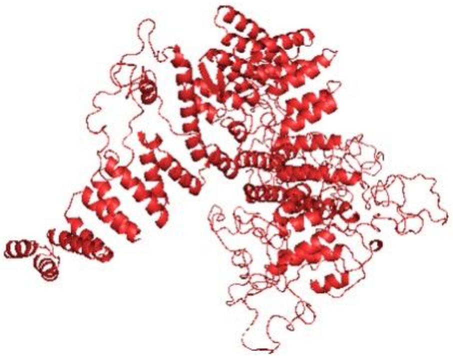

TbTau131

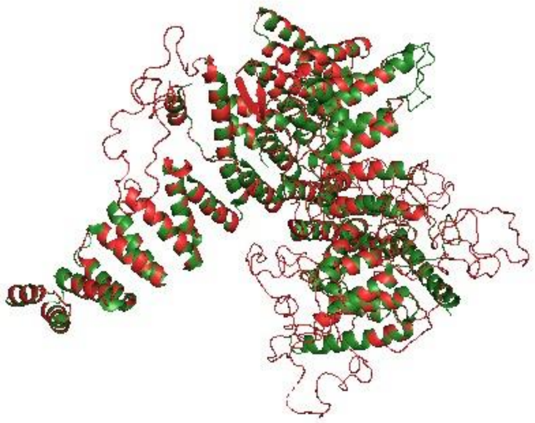

Merge

LmjF.12.0560

Annotated as: Hypothetical protein

Putative ortholog of *S. cerevisiae* **Tau131**. Probability: 100%. E-value: 1.3e-^{37}

```
Lm   33   SSSALHSTFEAHVTAAQNSFLRGDSQAAEHEAVQALRIRPTAH-LFALLAVIAESKGKFDQASDFRLLQAFLA-RDIVLW   110
Sc   120  KERVLDPEVAQLLSQANEAFVRNDLQVAERLFNEVIKKDARNFAAYETLGDIYQLQGRLNDCCNSWFLAAHLNASDWEFW   199
                        TPR 1                              TPR 2

Lm   111  EELLHDFMTQQLYYKSVVCLQRMSALEKNQERYRALQLQLADLFIGLGEIRRASNVLVPLWNSSRCRDFEVFALLSSLYF   190
Sc   200  KIVAILSADLDHVRQAIYCFSRVISLN--PMEWE-SIYRRSMLYKKTGQLARALDGFQRLYM-YNPYDANILRELAILYV   275
                 TPR 3                                   TPR 4

Lm   191  QLGKWASLQRLIESSVKHAFQLLPTAEVTGATTTAGETMGHGAEATAQVSSSTPGTEQQLSSPPPDSSISEQRVSKRVRF   270
Sc   276  DYDRIEDSIELYMKVFNANVERRE---------AILAA-----------LENALDSS------DEE--------------   315
                                  TPR 5

Lm   271  FGMDAGEDGAAAPVTADLTAPTAPMSLSPAEASLDEFRFDDFISPASPLSHPPSSLRPGDTASSTGAHRGGVVHQLYGER   350
Sc   316  -------------------------S-AA-EGEDADEK------E--P--------------------------------   328

Lm   351  IELRSSQDQKNFLTLVNVHAELLNEQGNFSDTVQ-LLRMAAGCLDVDLIELPPDLLVRLGVAYAFLGGVYEQPCRD-VFQ   428
Sc   329  ----------------------LEQDEDRQMFPDINWKKIDAKY--KCIPFDWSSLNILAELFLKLAV-----SEVDGIK   379
                                                                             TPR 6

Lm   429  HLVAH-C----------------------------------------SMEDYGDVLLDAAMSLQKVSMHAEAAQVYVTLA   467
Sc   380  TIKKCARWIQRRESQTFWDHVPDDSEFDNRRFKNSTFDSLLAAEKEKSYNIPIDIRVRLGLLRLNTDNLVEALNHFQCLY   459
                                                                             TPR 7

Lm   468  RYHQFVYDRVRRQVGVAEAELTLMQDTAPLDQVQAATDALKALKTEEADLKTVLAAALQGQAQSAYARELYEEAKEYAEC   547
Sc   460  DETFS-----------------------------------------------DVADLYFEAATALTRAEKYKEAIDFFTP   492
                                                                             TPR 8

Lm   548  VLQLEPT-HLQARLLLGRYYFYQRKDTAAAVEVLTPSKHEPALQRIQLGVFLVHVFQRAKRYVEAIALGVSIFELLLSSE   626
Sc   493  LLSLEEWRTTDVFKPLARCYK-EIESYETAKEFYELAIKSEPDD-LDIRVSLAEVYYRLNDPETFKHMLVDVVEMRKHQV   570
                                TPR 9                                TPR 10
```

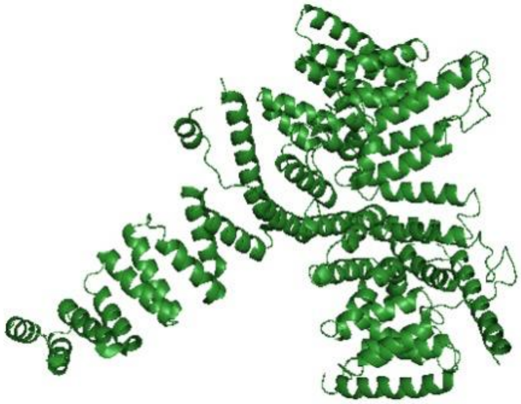

ScTau131

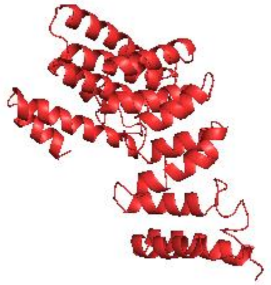

LmTau131

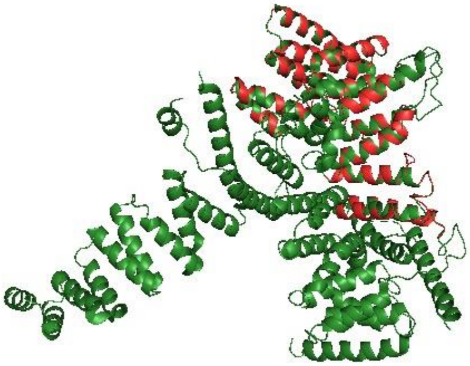

Merge

Tb927.11.4520

Annotated as: Hypothetical protein

Putative ortholog of *S. cerevisiae* **Tau138**. Probability: 78.91%. E-value: 13

```
Tb   474   SAVLDALNASPLRAMT-------LVDLVSV---VDRRTLGRVLPVLQRENRVTTTGVTLPQGRRLGVLVVAGVKLTDADR   543
Sc   557   RAILKVMNTIGGVAYLREQFYESVSKYMGSTTTLDKKTVRGDVDLMVESEKLGARTEPVS-GRKIIFLP----TVGEDAI   631
```

eWH domain

```
Tb   544   EELIKRH   550
Sc   632   QRYILKE   638
```

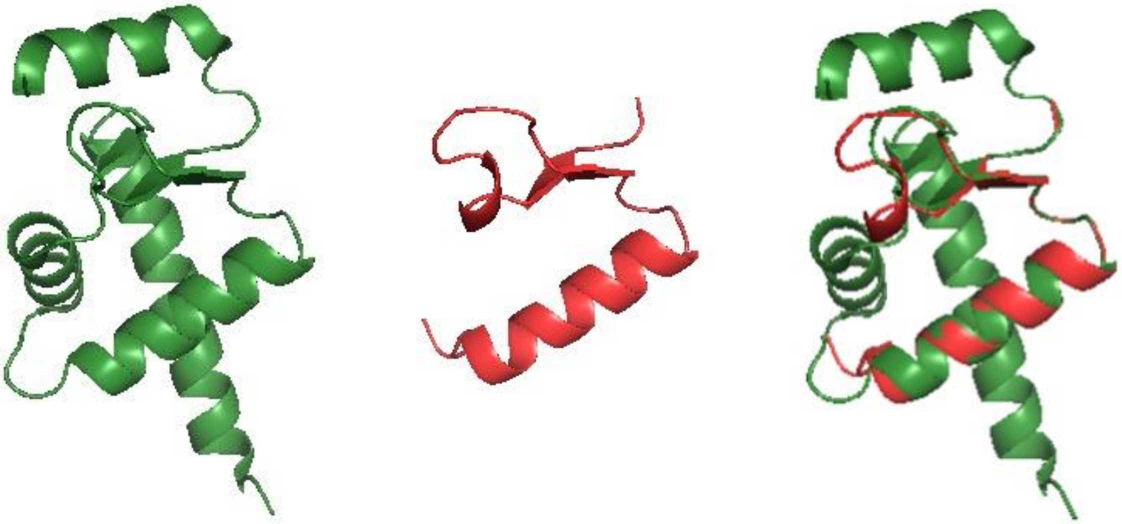

ScTau138 **TbTau138** **Merge**

LmjF.13.0270
Annotated as: Hypothetical protein
Putative ortholog of *S. cerevisiae* **Tau138**. Probability: 30.23%. E-value: 240

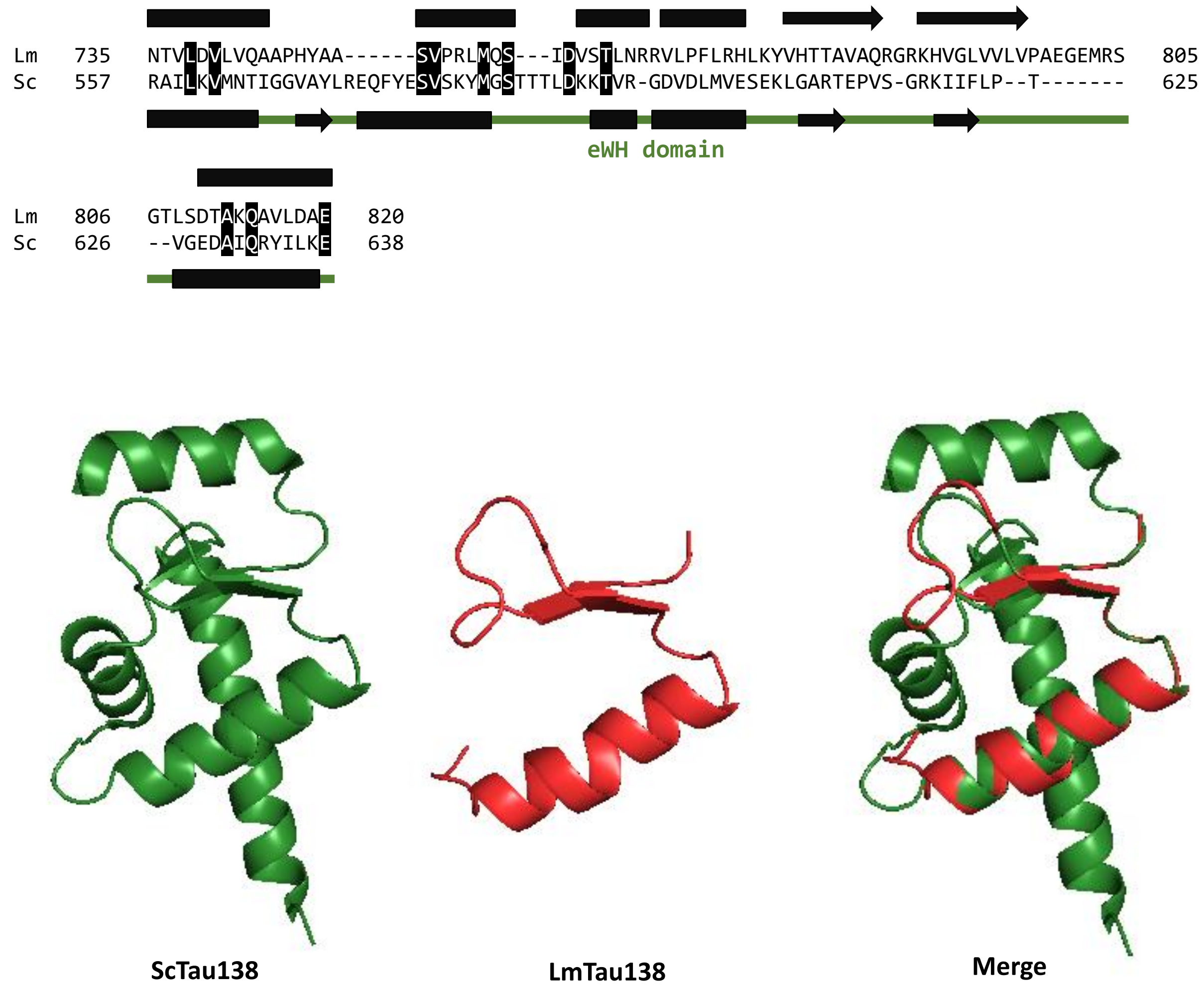

Supplemental Fig. S3. Sequence and structure analyses of the presumed orthologs of Tau55, Tau131 and Tau138 in *T. brucei* and *L. major*. The HHpred (https://toolkit.tuebingen.mpg.de/tools/hhpred) and Swiss-Model (http://swissmodel.expasy.org/interactive) programs were used to analyze the sequence and predicted three-dimensional structure of the three proteins. Conserved domains are indicated in the sequence alignments. For Tau131, the alignment shown corresponds to the first 10 TPRs (in the N-terminal region of the protein); the C-terminal region is not presented.
